# Spatiotemporal dynamics of tumor-associated neutrophils: bridging the gap between cancer progression and immunotherapy

**DOI:** 10.1186/s12943-026-02570-4

**Published:** 2026-01-26

**Authors:** Xiangyuan Chu, Junying Ma, Shihua Li, Meng Wang, Yu Tian, Chao Lv

**Affiliations:** 1 Department of General Surgery, Shengjing Hospital of China Medical University, Shenyang, Liaoning Province P. R. China; 2 Department of Obstetrics and Gynecology, Shengjing Hospital of China Medical University, Shenyang, Liaoning Province P. R. China; 3 Liaoning Province Center for Disease Control and Prevention, Sujiatun District, Shenyang, Liaoning Province P. R. China; 4 School of Electronic and Information Engineering, Liaoning Technical University, Xingcheng, Liaoning Province P. R. China

**Keywords:** Cancer immunotherapy, Spatiotemporal heterogeneity, Tumor microenvironment (TME), Tumor-associated neutrophils (TANs), Neutrophil extracellular traps (NETs)

## Abstract

Neutrophils, traditionally regarded as short-lived first responders of innate immunity, have emerged as pivotal regulators within the tumor microenvironment (TME). Recent advances reveal that tumor-associated neutrophils (TANs) and neutrophil extracellular traps (NETs) exhibit remarkable spatiotemporal heterogeneity, with their phenotypes and functions dynamically evolving across tumor developmental stages and anatomical niches. TANs and NETs display dual and context-dependent roles: they can promote tumor progression via immune suppression, angiogenesis, extracellular matrix remodeling, and metastatic niche formation, yet also exert anti-tumor functions through cytotoxicity and antigen presentation under specific microenvironmental cues. This review systematically dissects the spatial and temporal dynamics of TANs and NETs, emphasizing their molecular regulation by tumor-derived secretomes, chemokine gradients, hypoxia, stromal interactions, and inflammatory signaling networks. We further delineate the bidirectional crosstalk between TANs and other immune or stromal components that contributes to immune evasion and therapy resistance. Beyond mechanistic insights, we highlight emerging therapeutic strategies, ranging from chemokine axis blockade and phenotypic reprogramming to NETs inhibition and clearance, that hold promise for disrupting neutrophil-mediated tumor support. Finally, we advocate for the integration of cutting-edge spatial and single-cell multi-omics, imaging cytometry, and AI-assisted spatial modeling to enable high-resolution mapping of neutrophil dynamics in situ and to guide precision immunotherapy.

## Introduction

Neutrophils, as key effectors of the innate immune system, represent the first line of defense against pathogens and are rapidly recruited to sites of inflammation [1]. Once considered passive “foot soldiers” of host immunity, neutrophils are now acknowledged as dynamic and influential orchestrators within the tumor microenvironment (TME), endowed with substantial phenotypic and functional plasticity throughout tumor evolution. Early conceptual models categorized tumor-associated neutrophils (TANs) into anti-tumorigenic and pro-tumorigenic phenotypes, a dichotomy influenced in part by the presence of TGF-β, which drives neutrophils toward a tumor-promoting state [2]. However, mounting evidence indicates that TAN polarization is not fixed or binary, but occurs along a continuous spectrum shaped by tumor progression, metabolic cues (e.g., hypoxia, lactate accumulation, etc.), and therapeutic pressure [3–6]. This plasticity underlies the striking spatiotemporal heterogeneity observed in TAN populations. Across different tumor stages and anatomical niches, TANs display highly context-dependent behaviors and can exert either tumor-promoting or tumor-restraining functions [7, 8]. Collectively, recent findings highlight TANs as versatile and influential participants in cancer progression, whose dynamic states reflect the evolving ecological landscape of the tumor.

Neutrophil extracellular traps (NETs) are web-like structures composed of decondensed chromatin and granular proteins released by neutrophils, originally identified as a host defense mechanism against microbial pathogens [9, 10]. However, emerging evidence has fundamentally redefined this classical perspective. Recent studies have unveiled the dualistic roles of NETs in cancer biology. First, NETs facilitate tumor cell proliferation and metastasis by releasing matrix-degrading enzymes, such as metalloproteinases, which disrupt the ECM and generate “molecular conduits” that promote tumor cell invasion [11–15]. Additionally, NETs contribute to pre-metastatic niche formation by entrapping circulating tumor cells and shielding them from immune surveillance, thereby accelerating metastatic progression [16, 17]. Second, the antitumor effects of NETs appear to be highly context-dependent and vary according to tumor type and microenvironmental conditions [18]. Nonetheless, tumor-promoting functions of NETs are generally more prevalent. Recent findings demonstrate that NETs actively participate in shaping immunosuppressive TME by remodeling the ECM, promoting epithelial–mesenchymal transition (EMT), and impairing T-cell–mediated immune responses [19–21]. Elevated levels of NET-associated biomarkers have been correlated with poor clinical outcomes and may serve as prognostic indicators in multiple cancer types [22, 23]. Collectively, these insights highlight both the therapeutic promise and inherent complexity of targeting NETs in oncologic interventions.

The spatiotemporal heterogeneity of TANs is critical for elucidating their multifaceted roles in tumor initiation and progression. This heterogeneity reflects the fact that TAN functions are not static but dynamically shift in accordance with both tumor developmental stages and spatial localization. Temporally, TANs may exert robust anti-tumorigenic activity during early tumorigenesis, but progressively acquire pro-tumorigenic and metastasis-promoting phenotypes as the tumor advances [24, 25]. Among these processes, the recruitment of neutrophils from the circulation to tumor sites represents the initial step by which they participate in tumor immune regulation. This process is primarily driven by chemokines secreted by tumor cells, stromal cells, and other immune cells (e.g., CXCL1, CXCL2, CXCL8/IL-8) through their interactions with corresponding receptors (e.g., CXCR1, CXCR2) [26–28].

Once within the TME, neutrophils are further instructed by local cytokines, hypoxia, and metabolic signals to differentiate into TAN subsets with distinct functional properties. This results in functional heterogeneity and complex distribution patterns of TANs across different regions of the TME. For example, CD15⁺CD45⁺ neutrophils are preferentially enriched in perivascular regions, suggesting a role in vascular remodeling and immune cell trafficking [29]. In contrast, dcTRAIL-R1⁺ neutrophils primarily inhabit the glycolytic and hypoxic niches of the tumor core and are implicated in pro-angiogenic and immunosuppressive activities [30]. These spatially segregated TAN subpopulations and multifactorial landscape underscore the spatial complexity of neutrophil-mediated regulation in the TME. Emerging technologies such as single-cell RNA sequencing (scRNA-seq), spatial transcriptomics, and imaging cytometry have enabled unprecedented resolution in delineating the spatial distribution and phenotypic heterogeneity of TANs across distinct tumor compartments, including tumor cores, invasive fronts, and metastatic niches [31–33]. Notably, spatial proteomics has emerged as a powerful tool for characterizing the spatial architecture of the TME [34], will offer complementary insights into the localization and functional states of TANs.

Investigating the spatiotemporal dynamics of TANs and NETs provides critical insights into the intricate architecture of the tumor immune microenvironment and lays a conceptual foundation for the development of innovative therapeutic strategies targeting neutrophil-mediated regulation. This review centers on the spatiotemporal heterogeneity of TANs, with a focus on its cellular, molecular, and microenvironmental determinants. We begin by characterizing the major phenotypic subtypes, functional roles, and spatial distribution patterns of TANs, alongside the immunological and pathophysiological functions of NETs. We then delineate the mechanistic basis of their heterogeneity, emphasizing the dynamic interplay between TANs and a spectrum of TME components, including chemokine gradients, chronic inflammation, immune cell interactions, tumor-derived secretomes, abnormal vasculature, ECM remodeling, and hypoxic signaling, etc. Furthermore, we provide a systematic overview of emerging therapeutic strategies aimed at modulating TAN and NET biology, with particular attention to the translational potential and clinical feasibility of TAN-targeted interventions. Finally, we discuss the methodological advancements and analytical frameworks enabling high-resolution mapping of TAN distribution and function across spatial and temporal axes. We posit that elucidating the molecular circuitry underpinning TAN heterogeneity and harnessing this knowledge for targeted intervention may not only overcome existing therapeutic resistance but also redefine the paradigm of precision cancer immunotherapy.

## Spatiotemporal heterogeneity of TANs

### Neutrophil functions and subtypes in cancer

#### Neutrophil recruitment

The migration of neutrophils to tumor sites is a prerequisite for their functional engagement within the TME. This process is tightly orchestrated by a complex chemokine network that governs neutrophil trafficking and localization. Among these, the CXC chemokine family plays a pivotal role in dictating the spatial behavior of TANs. The functional versatility of the CXCL12–CXCR4 axis is rooted in its downstream signaling complexity. Upon ligand binding, CXCR4 activates heterotrimeric G-proteins, initiating Gα and Gβγ dissociation, GDP–GTP exchange, and subsequent activation of Ras, PI3K, and PLC pathways, culminating in intracellular calcium flux and neutrophil activation [35]. Beyond classical G-protein signaling, the axis also engages non-canonical pathways such as JAK–STAT3, Wnt/β-catenin, and CaMKII–CREB cascades [35, 36], contributing to the plasticity of TANs in different tumor niches. These findings emphasize the need to consider signaling pathway specificity and spatial context when targeting chemokine axes.

In addition to CXC chemokines, G-CSF critically influences neutrophil recruitment and functional polarization. Within metastatic niches, G-CSF promotes the mobilization of immature neutrophils from the bone marrow, skewing them toward immunosuppressive phenotypes that facilitate pre-metastatic niche formation [37–39]. Mechanistically, G-CSF activates the JAK–STAT3 pathway via CSF3R engagement on neutrophil progenitors, promoting proliferation and functional programming [40]. Notably, S100A8/A9 proteins and novel chemotactic mediators such as CXCL17 and ITGA3 also play significant roles in neutrophil migration [41]. These observations suggest that distinct chemokine combinations are dynamically activated at specific stages of tumor progression.

Therapeutic strategies targeting these chemokine networks have shown promise in preclinical studies. For instance, the CXCR2 inhibitor SB225002 effectively reduces immunosuppressive TAN infiltration and enhances CD8⁺ T cell activation in lung adenocarcinoma models [42]. Similarly, the CXCR4 antagonist plerixafor synergizes with PD-1 blockade to deplete myeloid-derived suppressor cells and augment T cell infiltration in ovarian cancer [43]. Nonetheless, incomplete mechanistic understanding remains a barrier to clinical translation. Future research employing spatial transcriptomics and intravital imaging to map neutrophil signaling at single-cell resolution will be essential to optimize combinatorial therapeutic strategies.

#### Multifaceted roles of TANs

Neutrophils represent the most abundant population of innate immune cells, characterized by significant spatiotemporal heterogeneity in their function and distribution within the TME. As the first responders to inflammation, neutrophils actively contribute to tumor dynamics, exhibiting dual regulatory roles that evolve over time and across spatial niches. Recent advancements in scRNA-seq and spatial transcriptomics have provided compelling evidence that neutrophil populations within tumors undergo dynamic phenotypic transitions. During early tumorigenesis, neutrophils predominantly localize at the tumor periphery, adopting an N1-like phenotype with anti-tumor properties. However, as tumor progresses, these cells gradually shift towards a pro-tumorigenic N2 phenotype, particularly within hypoxic, vascular and immune-suppressive niches [24, 25, 44, 45]. And in primary mouse pancreatic, skin and lung tumors, neutrophils are mainly enriched in CAF intensive areas, inducing NETs formation [46]. This temporal polarization process is largely driven by the dynamic cytokine gradients in the TME.

TANs are classically classified into two major subtypes: N1 (anti-tumorigenic) and N2 (pro-tumorigenic), a framework initially proposed by Fridlender et al. [1, 2]. TANs originate from myeloid progenitor cells and are recruited into the TME in response to various chemokines. Upon infiltration, they undergo context-dependent polarization under the influence of cytokine signaling: N1-TAN, a short-lived, functionally mature neutrophil subtype, is polarized by IFN-β, IL-1β, IL-8, and TNF-α, exhibiting potent cytotoxicity against tumor cells and enhancing CD8^+^ T cell activation; N2-TAN, a long-lived, immature neutrophil phenotype, is induced by TGF-β, IL-6, IL-8, and IL-17, promoting tumor growth, invasion, angiogenesis, and immune suppression [1, 8, 47–49]. Among these, IL-17 promotes the elevation of G-CSF levels, thereby polarized neutrophil, suppressing CD8⁺ T cells, and ultimately facilitating metastasis [50]. Notably, IL-8 exerts dual effects, contributing to both N1 and N2-TAN polarization depending on the local cytokine milieu and tumor stage, highlighting the plasticity of TANs in TME dynamics. Meanwhile, the functional outcomes of N1 and N2-TANs are distinct. N1-TANs exert anti-tumor effects through the production of ROS, myeloperoxidase (MPO), and cytotoxic mediators, which induce tumor apoptosis. N2-TANs drive tumor progression by shaping the metastatic microenvironment, promoting angiogenesis, immune suppression, and invasion via secretion of matrix metalloproteinase (MMP) 9, VEGF, and hepatocyte growth factor [1, 2, 8, 19]. A key feature of N2-TANs is their ability to generate NETs, which facilitate immune evasion, and promote metastasis. While NETs are predominantly tumor-promoting, there is emerging evidence that in certain geographic landscape contexts, TANs may also inhibit tumor migration and induce apoptosis, underscoring their functional plasticity. This topic will be discussed further in the next section.

The transition between N1 and N2 is not a simple dichotomy or static process, but rather a highly dynamic and context-dependent continuum shaped by spatial heterogeneity and tumor progression. Multiple studies have reported diverse neutrophil subpopulations exhibiting distinct density, surface markers, and transcriptional programs, suggesting functional diversification during tumor evolution [25, 30, 51, 52]. Intriguingly, in pancreatic cancer liver metastases, TANs exhibit a more aggressive transcriptional profile and enhanced immunosuppressive capacity compared to TANs in the primary tumor. This discrepancy is driven by NFE2, a transcription factor secreted by metastatic tumors, which reshapes TAN function to facilitate immune evasion and metastatic colonization [53]. This highlights the spatial specificity of neutrophil–tumor interactions in dictating cancer progression, which is further modulated by microenvironmental factors such as hypoxia, metabolic reprogramming, and immune suppression, etc [54–58]. These factors not only influence neutrophil recruitment and polarization but also shape their functional plasticity, thereby contributing to tumor evolution and therapeutic resistance.

#### Neutrophils in innate immunity

TANs exhibit significant spatiotemporal heterogeneity within the TME, assuming diverse functional roles depending on their localization and tumor stage. Understanding these differences in TAN distribution across tumor regions is critical for developing precision-targeted therapeutic strategies.

Broadly, TANs can exert either pro-tumorigenic or anti-tumorigenic effects. One of the most well-characterized tumor-suppressive mechanisms involves ROS production [1]. Mechanistically, these ROS activate H2O2-dependent Ca2^+^ channels in the plasma membrane of cancer cells, triggering calcium influx and subsequent cancer cell apoptosis [59, 60]. However, it is imperative to highlight that the effects of ROS on tumor cells and the immune system are concentration-dependent [61]. Several studies have demonstrated that ROS secreted by TANs can facilitate tumor progression. In peripheral blood, neutrophils exhibit higher maturity, with decreased c-Kit expression, significantly reduced mitochondrial function, and energy primarily derived from glycolysis. The stem cell factor (SCF) secreted by tumor cells can reprogram neutrophils through c-Kit signaling, so that neutrophils can still metabolize through mitochondria to produce ROS under glucose limitation conditions, maintain immunosuppressive activity, and promote tumor progression [62]. Conversely, a distinct subset termed tumor-entrained neutrophils, induced by primary tumors within pre-metastatic lungs, has been shown to restrain metastatic dissemination through hydrogen peroxide–mediated cytotoxicity rather than directly establishing a pre-metastatic niche [63]. In parallel, neutrophil-derived reactive nitrogen species exert tumoricidal effects by inducing oxidative DNA damage and apoptosis in malignant cells [1]. Moreover, tumor necrosis factor–related apoptosis-inducing ligand (TRAIL) released by TANs mediates programmed cell death in cancer cells, representing another mechanism through which neutrophils contribute to antitumor immunity [64].

However, the majority of TANs within the TME exhibit pro-tumorigenic and immunosuppressive properties, actively facilitating tumor progression and metastasis. Neutrophils recruited to TME are educated by tumor cells to obtain a unique phenotype that leads to the formation of TANs with specific functions. For example, IFIT1^+^ TANs colocalize with gastric cancer epithelial cells and are associated with epithelial interstitial transformation of gastric cancer. High infiltration of IFIT1^+^ TANs has been identified as a key feature in poorly cohesive carcinoma, where their upregulation promotes gastric cancer cell migration, invasion, and xenograft tumor growth [65]. Similarly, in RAS-driven zebrafish tumor models, neutrophils have been observed to promote tumor proliferation via prostaglandin E2 secretion [66]. Moreover, high levels of MPO^+^ neutrophils, as assessed by immunohistochemistry, correlate with poor clinical outcomes in Colon Adenocarcinoma [67]. In hepatocellular carcinoma, acetyl coenzyme A accumulation induces H3 acetylation, leading to CXCL1 overexpression, which recruits TANs and facilitates NET formation, ultimately driving metastasis [68]. The distinct functions of TANs are orchestrated by local molecular cues within the TME, which collectively drive tumor invasion, metastasis, and angiogenesis, while modulating immune responses. Dissecting these regulatory mechanisms offers critical insights into the ontogeny and functional programming of TANs, thereby unveiling novel perspectives and therapeutic targets for cancer diagnosis and treatment.

#### NETs, a hallmark feature of TAN activity in the TME

NETs, generated by TANs, also exhibit distinct spatial and temporal distribution patterns within the TME, reflecting the dynamic heterogeneity characteristic of TANs themselves. These chromatin-based structures contribute significantly to tumor progression through multiple mechanisms. In non-small cell lung cancer, for example, neutrophil infiltration is notably higher in squamous cell lung cancer than in adenocarcinoma, with squamous carcinoma-associated neutrophils exhibiting increased expression of NETs-specific markers, such as α-cleaved H3. The accumulation of these NETs fosters an immunosuppressive microenvironment that facilitates tumor immune evasion and disease progression [32]. Such complex intercellular interactions underscore the necessity of developing subtype-specific therapeutic strategies that precisely target NET-associated neutrophil dynamics, an area that warrants deeper mechanistic and translational investigation.

The prognostic significance of neutrophil extracellular traps has been validated in multiple studies and serves as a predictive marker for reduced overall survival. Clinically, NETs accumulation has been linked to poor patient outcomes across multiple malignancies. In Ewing’s sarcoma, intratumoral NET deposition is correlated with worse prognosis [23], while in metastatic colorectal cancer, elevated circulating NET markers are associated with an increased risk of tumor recurrence, further highlighting their potential as biomarkers for disease progression and relapse prediction [69]. Moreover, NETs have been implicated in driving EMT, a fundamental process that enhances tumor cell plasticity, invasiveness, and resistance to therapy, ultimately correlating with poorer clinical outcomes [20]. These findings emphasize the critical role of NETs in shaping the TME and reinforce their potential as therapeutic targets in cancer management.

Notably, NETs do not uniformly promote tumor progression. Under physiological conditions, neutrophils deploy NETs to entrap and neutralize pathogens, thereby limiting tissue damage and modulating immunity. For instance, NETs can enhance CD4⁺ T cell activation via upregulation of CD25, CD69, and ZAP70 phosphorylation [70]. Intriguingly, emerging evidence indicates that NETs may exhibit tumor-suppressive functions under specific microenvironmental contexts. In melanoma models, NETs have been shown to inhibit tumor cell migration and induce tumor cell necrosis in co-culture systems, suggesting a potential cytotoxic role [71]. However, contrasting studies report that increased densities of CD15⁺ TANs and H3Cit⁺ NETs correlate with metastatic dissemination in melanoma [72], underscoring the context-dependent and bidirectional nature of NETs function. This duality may arise from spatial and temporal heterogeneity: NETs formed in vasculature, at tumor margins, near dormant or proliferating cancer cells, or in distinct immune niches or ECM contexts may elicit divergent biological outcomes. For instance, NETs promote angiogenesis in gastric cancer [73], form physical barriers that exclude CD8⁺ T cells from tumors [17], and interact with regulatory T-cells (Tregs) to foster immunosuppression [74]. Collectively, these findings emphasize that elucidating the spatial context of NET formation is critical for understanding their paradoxical roles in cancer and for developing precision therapies targeting NET-mediated tumor regulation.

#### Reevaluating TAN subtypes: moving beyond the N1/N2 paradigm

The functional and phenotypic diversity of TANs is shaped by multiple cues within distinct TME. Identifying specific tumor-promoting TAN subpopulations represents an essential step toward refining neutrophil-targeted cancer therapies. However, the conventional binary classification of TANs into N1 (anti-tumorigenic) and N2 (pro-tumorigenic) subtypes fails to capture the extensive heterogeneity observed across different cancers, disease stages, and spatial niches. This dichotomous framework oversimplifies the dynamic continuum of neutrophil states, limiting our understanding of TAN evolution and therapeutic potential, and underscoring the need for a more nuanced classification system that integrates molecular, functional, and spatial dimensions.

In non–small cell lung cancer, for instance, five distinct TAN subtypes have been delineated: TAN-0 (antigen-presenting), TAN-1 (intergenic), TAN-2 (pro-tumor), TAN-3 (classical), and TAN-4 (pro-inflammatory), revealing a complex spectrum of neutrophil phenotypes and context-dependent functions [75].This functional heterogeneity inspired the development of a prognostic model using six neutrophil differentiation-related genes (CTSZ, PLAUR, NME2, NPM1, EIF3E, and PPIA). Patients with a low-risk signature, characterized by high CTSZ and low EIF3E, PPIA, and PLAUR expression, exhibited improved overall survival and enhanced responses to immunotherapy, reinforcing the clinical relevance of TAN subtyping [75]. While these findings highlight intratumoral diversity within a single cancer type, broader integrative analyses have revealed conserved neutrophil programs across malignancies.

Extending beyond tissue-specific studies, pan-cancer single-cell multi-omic analyses have identified three evolutionarily conserved terminal TAN states: T1 (immature, dcTRAIL-R1-CD101-), T2 (mature, dcTRAIL-R1-CD101+), and T3 (terminally differentiated, dcTRAIL-R1+). Both T1 and T2 neutrophils undergo deterministic reprogramming into a terminal T3 phenotype through irreversible epigenetic, transcriptional, and proteomic remodeling, independent of their initial maturation status [30]. The conservation of this T3 signature across mouse and human tumors, and its association with poor prognosis, argue for a classification framework that transcends the simplistic N1/N2 dichotomy and incorporates spatially defined terminal neutrophil states within the TME.

Building on these lineage-resolved findings, spatially resolved analyses have revealed that TAN phenotypes are tightly coupled to anatomical niches. Peritumoral and perivascular regions are enriched for distinct neutrophil subsets, whereas dcTRAIL-R1⁺ TANs preferentially occupy hypoxic–glycolytic tumor cores, where they display pronounced pro-angiogenic and immunosuppressive features [30]. The tumor invasive front represents another spatially distinct immune ecological niche enriched in TAN subtypes with pronounced pro-tumorigenic properties. This region, characterized by dense tumor infiltration and immune clustering, supports the emergence of multiple functionally specialized TAN subsets. For instance, CD11b⁺CD15⁺CD10⁺ neutrophils induce EMT and tumor budding, correlating with adverse clinical outcomes [25]. Similarly, in a pancreatic cancer liver metastasis model, six transcriptionally and functionally distinct TAN subpopulations (CXCL1⁺, CCL3⁺, HSPA6⁺, RPS19⁺, ISG15⁺, and S100A12⁺) were identified, each associated with specific molecular programs linked to invasion and metastatic progression [53]. In hepatocellular carcinoma, urokinase-type plasminogen activator receptor (uPAR)⁺ TANs inhibit CD8⁺ T cell infiltration by downregulating chemokines CXCL9 and CXCL10 while simultaneously recruiting and polarizing M2 macrophages via the CCL2–CCR2 axis, thereby reinforcing immunosuppression and contributing to resistance to PD-1 blockade [76].

Collectively, these findings illustrate that TAN heterogeneity arises from the intersection of molecular programs, differentiation trajectories, and spatial context. Niche-dependent plasticity and deterministic cell-state transitions jointly shape TAN function, explaining how neutrophils progressively acquire protumor phenotypes within specific microenvironmental landscapes. This conceptual framework underscores the need for refined classification systems that integrate molecular signatures, functional states, and spatial localization to capture the true complexity of TAN biology.

Despite these advances, most studies continue to define TAN subsets based on a limited set of phenotypic markers, resulting in substantial variability across tumor types and experimental conditions. This methodological inconsistency hampers cross-study comparability and impedes the development of standardized, clinically relevant neutrophil-targeting approaches. Therefore, establishing a unified nomenclature that integrates maturation state, molecular programs, and spatial positioning is essential to resolve TAN diversity, facilitate data harmonization, and enable the design of precision neutrophil-directed therapies. Moving beyond the oversimplified N1/N2 paradigm will not only deepen mechanistic understanding of TAN biology but also accelerate the clinical translation of neutrophil-targeted interventions.

### Heterogeneity in spatial dimension

#### Microenvironmental influence on TAN distribution

The spatiotemporal dynamics of TANs are evident in tumor progression. Across cancer types, the extent and topology of TAN infiltration vary with tumor‑specific inflammatory programs and evolve during disease progression in response to dynamic chemokine gradients, metabolic stress, and hypoxia [30–32].

The degree and pattern of TAN infiltration vary substantially across tumor types. Higher levels of TAN accumulation have been reported in lung and renal cell carcinomas, moderate levels in gastrointestinal tumors, and relatively lower infiltration in other malignancies [31]. Even within a single organ system, TAN distribution exhibits subtype-specific variation. In non–small cell lung cancer, neutrophil infiltration is significantly greater in immune-excluded squamous cell carcinoma than in lung adenocarcinoma, with the density of intratumoral neutrophils being approximately twice as high in the former [77]. These differences are largely orchestrated by tumor-intrinsic inflammatory signaling profiles, whereby inflamed TMEs display enhanced neutrophil-recruiting capacity. Importantly, TAN localization is not static but evolves throughout tumor progression, dynamically reshaped by cytokine gradients, nutrient deprivation, and oxygen fluctuations.

TANs preferentially accumulate within perivascular and metastatic niches, where they engage in reciprocal interactions with stromal and tumor cells to sustain immunosuppression and metastatic dissemination. In perivascular regions, s3-type CAFs co-localize with TANs and recruit them via CXCL12–CXCR4 signaling while modulating their immunophenotype through ANXA1–FPR1 and APP–CD74 axes [78]. In the context of metastatic niches, particularly within pulmonary metastases, TANs exhibit distinct spatial positioning, closely associated with proliferative ApoE⁺Ki67⁺ tumor cells. These neutrophils display high expression of proliferation and activation markers (Ly6G, ApoE, Ki67), S100A4 and Thbs1 to construct an immunosuppressive network that promotes metastatic colonization [79]. Similarly, a tumor-elicited vascular-restricted TAN population (Ly6G^high^Ly6C^low^) has been described, characterized by robust NET-forming potential but limited extravasation. These intravascular TANs aggregate within abnormal tumor vessels, occlude blood flow, and drive pleomorphic necrosis. Inhibition of NETs formation attenuates both necrosis and metastasis, directly linking a spatially restricted TAN phenotype to metastatic risk [80].

TANs also display significant phenotypic and functional heterogeneity across tumor types, reflecting the influence of distinct microenvironmental cues on their polarization and spatial behavior. CD177, a glycosylphosphatidylinositol-anchored glycoprotein expressed on neutrophils and their precursors, is upregulated during inflammation to facilitate neutrophil trafficking and modulate the release of inflammatory mediators and NET formation. In colitis-associated and sporadic colorectal cancers, infiltration of CD177⁺ TANs has been associated with improved overall and disease-free survival, whereas in pancreatic ductal adenocarcinoma, increased CD177⁺ TAN presence correlates with poor prognosis [81, 82]. Similarly, immune composition varies substantially in brain metastases from distinct primaries: melanoma-derived metastases are dominated by CD4⁺ and CD8⁺ T cells, while breast cancer brain metastases exhibit higher neutrophil abundance [83]. Transcriptomic profiling has further identified diverse TAN subsets. HLA-DR⁺CD74⁺ TANs are enriched in non-small cell lung cancer, bladder, and ovarian cancers, whereas their infiltration is reduced in renal cell carcinoma and oral squamous cell carcinoma. In contrast, VEGFA⁺SPP1⁺ TANs are enriched in renal cell carcinoma and gastric cancer [31]. These findings underscore the spatiotemporal complexity of TANs and their regulation by the TME, though the precise molecular mechanisms remain to be fully elucidated.

Chemokine gradients play a decisive role in directing neutrophil positioning. For instance, neutrophil positioning in lung tissue is critically dependent on the CXCL12–CXCR4 axis, whereas other organs such as the small intestine employ alternative molecular pathways to imprint tissue-specific phenotypes on neutrophils [28]. Within the TME, immune cells, chemokines, and tumor cell-derived factors collectively shape neutrophil infiltration patterns. Macrophage-derived CCL3, for example, recruits TANs into the TME and facilitates their direct engagement with tumor cells [84]. Moreover, heterogeneous microenvironmental characteristics drive spatially distinct neutrophil behaviors. In gallbladder cancer, neutrophil density is markedly higher at hepatic invasion sites compared to localized tumors or normal tissues, with preferential accumulation at the invasive front relative to the tumor core. Mechanistically, KRT17⁺ tumor cells have been shown to induce neutrophil uptake of oxidized low-density lipoprotein through direct cell–cell contact, triggering metabolic reprogramming that facilitates neutrophil infiltration. Concurrently, hypoxia, glycolytic metabolism, and signaling pathways such as CCL4L2–VISTA and MIF–CD74 collectively establish a molecular network that governs the spatiotemporal recruitment and functional remodeling of TANs [85].

Spatial interactions between TANs and other immune cells further modulate their roles in the TME. Co-localization of TANs and TAMs has been observed in multiple cancer types; in intrahepatic cholangiocarcinoma, TAN–TAM interactions enhance tumor cell proliferation and invasion [33]. In gliomas, TANs acquire an inflammatory phenotype under the influence of the brain-specific TME, contributing to immunosuppression [29]. Notably, recruitment of TANs by IL-8 and G-CSF is often accompanied by the spatial proximity of HLA-DR⁺ antigen-presenting neutrophils to anti-tumor T cell subsets, a phenomenon associated with favorable clinical outcomes [31].

Collectively, spatial, molecular, and immunological determinants interact to orchestrate TAN distribution and functional adaptation within tumors. This dynamic and context-dependent network underscores the necessity of integrating spatial biology with molecular and immunological profiling to fully elucidate TAN-mediated tumor regulation. A deeper understanding of these microenvironmental influences will be pivotal for developing precision immunotherapeutic strategies targeting the spatiotemporal heterogeneity of TANs.

#### Prognostic implications of TAN spatial localization

The spatial localization of TANs within tumors has emerged as a critical determinant of patient prognosis. Recent studies have revealed that the differential localization of TANs within distinct TME subregions exhibits significant correlations with their functional polarization and clinical outcomes (Table 1). This spatial perspective provides novel insights into the dual roles of neutrophils in tumor progression and therapy response.

**Table 1:** Spatial distribution of TAN subsets within the tumor microenvironment and their associations with prognosis and therapeutic response

TAN subtypes		Cancer	Location			Prognosis and Targets	Reference
CXCR1^+^		EGFR-mutant Non-small cell lung cancer	Tumor core			Associated with poor prognosis; infiltration of CXCR1⁺ neutrophils correlates with the efficacy of third-generation EGFR-TKIs in patients harboring EGFR mutations.	[86]
VEGFA^+^SPP1^+^		Pan-cancer	vitamin metabolism and glucose metabolism-specific			Associated with unfavorable prognosis; reducing VEGFA⁺ SPP1⁺ neutrophil infiltration may relieve their suppression of T cells and thereby improve responses to PD-1/PD-L1 inhibitors.	[31]
HLA-DR^+^		Pan-cancer	Tumor-specific			Associated with favorable prognosis; a leucine-enriched diet can activate HLA-DR⁺ neutrophils in vivo, increase their proportion within the TME, and promote CD8⁺ T-cell infiltration.	[31]
CD15^+^ CD45^+^		Glioma	Perivascular regions			Associated with unfavorable prognosis; Recruits neutrophils into the TME, releases NETs, and suppresses T cell function	[29]
CD10^+^		CRC	Invasive front			Associated with poor prognosis; correlated with EMT of tumor cells and formation of tumor budding.	[25, 87]
IL-17a ^+^		GC	Invasive front			Associated with poor prognosis; predicts shorter disease-free survival and disease-specific survival. Neutralizing IL-17a with antibodies or inhibiting JAK2/STAT3 with AG490 reverses TAN-induced migration, invasion, and EMT in GC cells.	[88]
Ly6G^+^Ly6C^−^		Breast cancer	Surrounding areas of tumors with polymorphic necrosis			Associated with poor prognosis; Obstruction of downstream vessels induces hypoxia, which triggers EMT of cancer cells around necrotic zones and promotes metastasis.	[80]
dcTRAIL-R1^+^CD101^−/+^		PDAC	Intratumoral niches characterized by hypoxia and high glycolytic activity			Associated with poor prognosis; Promotes tumor angiogenesis and supports tumor growth.	[30]
CD66b^+^		Cervical cancer	Peritumoral and stromal regions			Associated with worse relapse-free survival.	[89]
N2-type^*^		HCC	Peritumoral and stromal regions			Associated with inferior OS.	[5, 90]
Ly6G^+^Ly6C^+^		Kras-driven mouse model of lung cancer	Tumor margin or areas in direct contact with tumor cells			Associated with therapy resistance; Reduces T cell homing and prevent anti-PD1 efficacy by altering angiogenesis, leading to hypoxia and Snail stabilization in tumor cells.	[91]
FCGR3B^+^S100A9^+^		Gallbladder cancer liver invasion	Invasive front at the tumor liver boundary			Associated with tumor invasion; Co-localizes with regulatory T cells and M2-like tumor-associated macrophages; exhibits deeper penetration toward the tumor core.	[85]
stromaTANs^*^		CRC	Tumor stroma			Significantly associated with lymph-node metastasis and tumor deposits; may promote invasion and metastasis.	[92]
TNFα^+^ICAM-1^+^ CD95^+^		CRC	Early invasive edge			Exhibits anti-tumor activity.	[93]
CD66b^+^		CRC	—			Associated with favorable prognosis; co-infiltration with CD8⁺ T cells markedly enhances the prognostic value of the latter.	[94]
CD66b^+^		CRC	Invasive front			Expression at the invasive front, tumor center, and intraepithelial regions correlates with adverse prognosis.	[95, 96]
CTCF^+^		CRC	Hypoxic tumor regions			Associated with poor prognosis and resistance to immunotherapy. CTCF^+^ TANs suppress T-cell immunity, facilitate EMT, and contribute to CRC progression and metastasis	[97]

Abbreviation: *TME* tumor microenvironment, *TAN* tumor-associated neutrophils, *NETs* neutrophil extracellular traps, *CRC* colorectal cancer, *EMT* epithelial mesenchymal transition, *GC* gastric cancer, *PDAC* pancreatic ductal adenocarcinoma, *HCC* hepatocellular carcinoma

^*^The article does not clearly specify the specific manifestations of neutrophils

Across multiple cancer types, intratumoral accumulation of TANs, particularly within tumor cores, has been consistently associated with poor prognosis and resistance to therapy [25, 86, 98–100]. This adverse impact likely reflects the establishment of an immunosuppressive milieu in which cytokines such as TNF-α and GM-CSF drive neutrophil polarization toward a tumor-promoting phenotype, suppress CD3⁺ T cell activity, and facilitate immune evasion [25, 101]. These cytokines also induce PD-L1 expression on neutrophils in hepatocellular carcinoma, reinforcing local immune suppression [90].

By contrast, TANs localized at the tumor invasive front or in peritumoral stroma are frequently linked to enhanced tumor aggressiveness and unfavorable outcomes. A high density of CD66b^+^ neutrophils in the peritumoral or stromal compartments, rather than within tumor nests, predicts reduced recurrence-free survival in cervical cancer [89]. Similarly, in hepatocellular carcinoma, neutrophils are predominantly located in the peritumoral stroma and similarly correlate with poor prognosis [5]. Spatial analysis in colorectal cancer further reveals that CD11b⁺CD15⁺CD10⁺ TANs preferentially accumulate at the invasive front of tumors, where they are strongly associated with TGF-β expression and the formation of tumor buds, which are small clusters of cells believed to have undergone EMT and are recognized as hallmarks of aggressive disease and poor clinical outcome [25, 87]. Collectively, these observations underscore the critical influence of spatially distinct TME niches in dictating TAN phenotype and function, while highlighting the therapeutic potential of exploiting TAN spatial heterogeneity as a foundation for precision intervention.

Building on this concept, emerging and next-generation strategies are being developed to selectively modulate TAN activity in a spatial- and phenotype-specific manner. For instance, developing depolarization strategies for immunosuppressive TANs in the tumor core or designing targeted blockade approaches against pro-metastatic TANs at the invasive front may open new avenues for improving therapeutic outcomes in cancer [102–104]. CXCR2 inhibitors can target and deplete pro-tumorigenic TANs enriched in tumor cores, while nanocarrier-based platforms enable site-specific drug delivery at the invasive front, where TANs potentiate EMT and local dissemination [1, 42, 105]. Moreover, therapeutic strategies combining neutrophil-targeting agents with immune checkpoint inhibitors (ICIs) have demonstrated synergistic effects. For instance, dual inhibition of CXCR1/2 signaling alongside PD-1 blockade not only effectively reduces intratumoral neutrophil burden but also enhances CD8⁺ T cell infiltration and activation [43, 106, 107].

As spatial omics technologies continue to evolve, high-resolution mapping of TAN microlocalization and dissection of their spatiotemporal interaction networks with stromal and immune populations are poised to reshape our understanding of TAN biology [108]. Notably, CTCF^+^ TANs enriched in the hypoxic tumor regions exhibit enhanced migratory capacity and IL-1β secretion, which are associated with a poor prognosis and resistance to immunotherapy [97]. Integrating such spatially resolved insights into clinical frameworks will be instrumental for translating TAN heterogeneity into prognostic biomarkers and for designing next-generation spatially targeted immunotherapies.

#### TAN interactions with tumor components

The spatial behavior of TANs plays a critical role in regulating tumor progression. Emerging evidence indicates that TANs construct spatially distinct regulatory networks through interactions with specific TME compartments, providing essential insights for the development of localized, microenvironment-targeted therapeutic strategies.

Recent studies in CRC demonstrate a striking location-dependent dichotomy in TAN behavior. Intratumoral TANs correlate with the number of resected lymph nodes, the degree of fibrosis, and receipt of neoadjuvant therapy, and even show a trend toward improved 3‑year disease‑free survival [92]. In contrast, stromal TANs are significantly associated with lymph‑node metastasis and tumor deposits, suggesting their involvement in invasion and dissemination [92]. Interestingly, in early stage of CRC, tumors overexpress neutrophil‑attracting chemokines, recruiting TANs to both the invasive margins and tumor centers. At the invasive front, TANs exhibit an antitumor phenotype characterized by high TNF-α, ICAM-1, and CD95 expression; TAN-derived TNF-α induces tumor-cell apoptosis, while CD95–CD95L engagement between TANs and cancer cells triggers apoptosis in both populations [93].

Consistent with this spatial dichotomy, high CD66b⁺ neutrophil infiltration has been linked to favorable prognosis in CRC, particularly when co-infiltrating with CD8⁺ T cells, which markedly amplifies their prognostic value [94]. Conversely, low neutrophil density at the tumor invasive front independently predicts poor outcome in early-stage colon cancer [95], whereas high intratumoral CD66b⁺ neutrophils are positively associated with advanced tumor stage, distant metastasis, and shortened survival [96]. These findings collectively underscore a spatially contingent biology in which the localization of TANs, whether within tumor nests or at stromal and marginal zones, carries opposing prognostic implications.

At tumor invasive fronts, TANs drive tumor progression, invasion, and metastasis through multidimensional mechanisms. Single-cell spatial transcriptomic studies reveal that Ly6G^+^Ly6C^+^ neutrophils form tertiary lymphoid structure-like architectures in these areas, promoting tumor aggressiveness via spatially coordinated triple mechanisms: immune exclusion, vascular remodeling, and Snail expression regulation [91]. Similar spatial specialization patterns have been observed in other tumors. For example, PD-L1^+^ neutrophils at the invasive margin of hepatocellular carcinoma suppress T cell function through the PD-1/PD-L1 axis [90]. The CD11b⁺CD15⁺CD10⁺ subset at colorectal cancer frontiers induces EMT through pro-inflammatory factor secretion, promoting prognostically unfavorable tumor budding [25]. Furthermore, IL-17α⁺ TANs within gastric cancer microenvironments activate EMT via the JAK2–STAT3 signaling pathway [88], suggesting that TAN-mediated regulation at invasive fronts may represent a conserved mechanism across multiple tumor types, offering valuable cross-cancer biological insights.

Beyond tumor invasion, the spatial heterogeneity of TANs also provides niche support for tumor recurrence. In pancreatic ductal adenocarcinoma liver metastasis models, chemotherapy-surviving residual tumor cells recruit TANs to metastatic sites through CXCL1/2 signaling, forming a GAS6-AXL-dependent regenerative microenvironment [109]. This therapy-induced spatial reprogramming of TANs highlights the dynamic nature of tumor–neutrophil interactions during the formation of drug-resistant niches. Significantly, the spatial positioning of TANs shows topological correlation with their immunomodulatory functions. For instance, infiltrating TANs, via activation of the ANXA2–TLR2–MYD88 axis, generate ARG1-mediated L-arginine depletion zones, forming localized immune barriers that restrict lymphocyte infiltration [110]. Hepatocellular carcinoma studies further demonstrate that CCL4⁺ TANs spatially synergize with PD-L1⁺ subsets through macrophage recruitment and T cell suppression, constructing multicellular immunosuppressive complexes [51]. These findings indicate that the immunoregulatory functions of TANs are highly spatially structured, facilitating tumor immune escape through the formation of regional immunosuppressive microenvironments.

Collectively, these studies reveal that TANs exert profound influence on tumor development, invasion, and recurrence through spatially specialized functional modalities. Future advances in spatial omics and real-time imaging technologies are anticipated to further unravel the compartmentalized regulation of TANs, laying theoretical foundations for developing innovative therapies targeting neutrophil spatial heterogeneity.

#### NETs in driving tumor dissemination

NETs contribute to malignant progression by orchestrating a multidimensional regulatory network, with their biological effects exhibiting distinct spatial characteristics. The localization patterns and tissue-specific deployment of NETs across distinct TMEs critically influence their biological effects. Rather than exerting uniform functions, NETs interact with a variety of TME components to construct pro-metastatic niches, promote stromal remodeling, and modulate immune cell dynamics, thereby facilitating tumor progression.

During the early stages of metastasis, NETs coordinate the formation of supportive niches at both primary and distant sites through dual mechanisms. In primary hepatocellular carcinoma models, NETs selectively reprogram resident hepatic stellate cells via metabolic activation, converting them into CAFs with pro-metastatic potential. This NET–stellate cell axis functions as a pivotal node in promoting intrahepatic metastasis by inducing vasculogenic mimicry and fostering an immunosuppressive microenvironment [111]. Spatial analysis of NETs distribution in metastatic liver tissues may reveal actionable windows for preventive or perioperative therapeutic interventions.

At the circulating tumor cell colonization stage, NETs demonstrate multimodal pro-metastatic mechanisms. Lung metastasis models show that NETs facilitate physical retention of circulating tumor cells through platelet-tumor cell aggregate entrapment [112]. Simultaneously, NETs induce cytoskeletal reorganization via the CCDC25-integrin-linked kinase signaling axis, enhancing tumor cell motility and supporting transendothelial migration [16]. Together, these processes enable coordinated regulation of tumor cell extravasation and distal organ colonization.

#### Distribution and functional impact of NETs

The density and spatial distribution of NETs have been increasingly recognized as critical determinants of tumor progression and patient prognosis. Given the multifaceted role of NETs in promoting tumor cell invasion, immune suppression, and metastatic niche formation, their accumulation in various anatomical compartments reflects the biological aggressiveness of the malignancy. Clinical studies have shown that elevated levels of circulating NET markers, including citrullinated histone (H3Cit) and MPO–DNA complexes, are detected in patients with lung and colorectal liver metastases, and are associated with advanced disease stages and poor prognosis [111]. These circulating NETs may serve as non-invasive biomarkers for metastatic burden or systemic NET activation, offering potential utility in real-time disease monitoring and risk stratification.

Importantly, NETs are not restricted to the circulation. Increased NET accumulation has been documented at primary tumor sites, in peripheral blood, and within metastatic lesions, suggesting that tumors can actively induce NET formation in both local and distant sites [113]. This phenomenon reflects a possible tumor-instructed systemic priming of neutrophils, resulting in spontaneous NET production and continuous reinforcement of a pro-metastatic environment. The identification of NETs in these spatially distinct compartments not only provides insight into tumor-induced innate immune reprogramming, but also reinforces the concept that NET burden may represent a surrogate marker of disease aggressiveness, with implications for prognosis, treatment response, and therapeutic decision-making.

### Temporal heterogeneity

#### Evolution over tumor progression

Recent studies have demonstrated that TANs exhibit pronounced spatiotemporal heterogeneity throughout tumor progression, characterized by dynamic phenotypic transitions and functional remodeling that follow conserved evolutionary trajectories across diverse cancer types. This plasticity is governed not only by tumor stage but also by the progressive spatial reprogramming of the TME, ultimately driving the convergent emergence of pro-tumor neutrophil phenotypes.

In early tumorigenesis, TANs are typically scarce but progressively accumulate as tumors advance. This temporal accumulation is particularly evident in breast cancer, where TANs in advanced stages enhance the expression of proliferation markers such as Ki67 and invasion-related molecules through direct cell–cell contact, thereby reinforcing malignant potential [84]. Similarly, in melanoma, the frequency of precursor or immature neutrophil subsets positively correlates with disease stage, whereas subsets expressing mature markers (CD101, CD10, CD16) show an inverse relationship. This temporal shift in subset composition suggests that the relative abundance of immature neutrophils may serve as a biomarker for disease progression, with enrichment of precursor subsets indicating advanced disease [114].

Temporal evolution is also reflected in neutrophil density and function. Single-cell analyses reveal that mature, high-density neutrophils gradually convert into low-density phenotypes, which preferentially accumulate in advanced-stage tumors [39]. This density shift likely parallels functional polarization. For example, in non-small cell lung cancer, spatial interactions between TANs and CD4⁺ or CD38⁺ T cells are progressively replaced by immunosuppressive CD163⁺ macrophages, suggesting the formation of stage-specific suppressive niches [75]. As previously described, in pancreatic cancer models, T1 and T2 neutrophils undergo deterministic differentiation into T3 TANs, which localize to hypoxic, glycolytic tumor regions and promote angiogenesis and tumor expansion. These terminally differentiated T3 cells exhibit distinct epigenetic and proteomic profiles, reflecting irreversible pro-tumor reprogramming [30].

This temporal plasticity of TANs presents dual therapeutic implications. On one hand, early-stage interventions that prevent neutrophil recruitment into the TME may interrupt the formation of pro-tumor phenotypes. On the other, targeting terminally differentiated TANs through reversal of their epigenetic memory or metabolic reprogramming, may offer opportunities to restore anti-tumor immune responses and reshape the immunological landscape of advanced tumors [30].

#### Limitations in studying temporal heterogeneity

Understanding the temporal heterogeneity of TANs is crucial for the development of more effective cancer therapies, as it enables the formulation of stage-specific strategies to selectively target distinct phases of TAN activation and function. However, current studies on TAN temporal dynamics remain constrained by substantial technical and methodological limitations. One of the primary challenges lies in the intrinsic biological properties of neutrophils: their low RNA content, limited transcriptional activity, and short half-life place considerable demands on experimental precision and sample handling [115]. Moreover, since TAN plasticity is a dynamic and evolving process, temporal resolution necessitates repeated sampling across multiple time points, significantly increasing experimental complexity and logistical burden. In clinical settings, continuous longitudinal sampling is often infeasible due to ethical concerns and procedural limitations. This poses a particular challenge when attempting to capture the phenotypic and functional evolution of TANs over time within the TME.

While organoid models have emerged as valuable tools for recapitulating tumor–immune cell interactions, limitations persist in sustaining functional immune components over extended culture periods. For instance, in a study involving advanced renal clear cell carcinoma, organoid platforms were employed to assess the ratio of CD8⁺ T cells within CD3⁺ populations and the activation state of CD69⁺ T cells under immunotherapy conditions. However, the immune cell and stromal components declined over time, reflecting the challenge of maintaining immune viability and cellular dynamics in ex vivo systems [116]. At present, the most widely used approaches for studying temporal changes in TANs rely on sequential sampling combined with high-dimensional analyses such as flow cytometry or single-cell multi-omics [30, 117]. However, these methods inherently provide static snapshots of dynamic cellular states, limiting the temporal continuity of observation. Recent advancements in live-cell imaging and intravital microscopy offer promising avenues to overcome this limitation. For example, in a murine model of cytokine storm, researchers employed real-time imaging to monitor neutrophil-derived extracellular vesicle production, exocytosis dynamics, and interactions with macrophages within living tissues, providing a more accurate depiction of the dynamic immune landscape [118].

Looking forward, continuous technological innovations are expected to expand our capacity to capture the real-time dynamics of TANs and their interactions with other TME components. As novel platforms, such as spatially resolved live-cell imaging, multi-dimensional organoid systems, and in situ barcoding, continue to evolve, they will enable more comprehensive and mechanistically precise investigations into the temporal behavior of neutrophils. These advances will help overcome current technical bottlenecks and ultimately inform the development of more effective, temporally guided immunotherapeutic strategies. In the interim, careful consideration must be given to experimental design, including the selection of sample types, optimal collection time points, and the most appropriate analytical technologies. Balancing research objectives with methodological feasibility will be crucial for maximizing the translational impact of TAN-focused studies in dynamic tumor ecosystems.

## Molecular and environmental drivers of TAN heterogeneity in spatiotemporal landscape

**Fig. 1:**
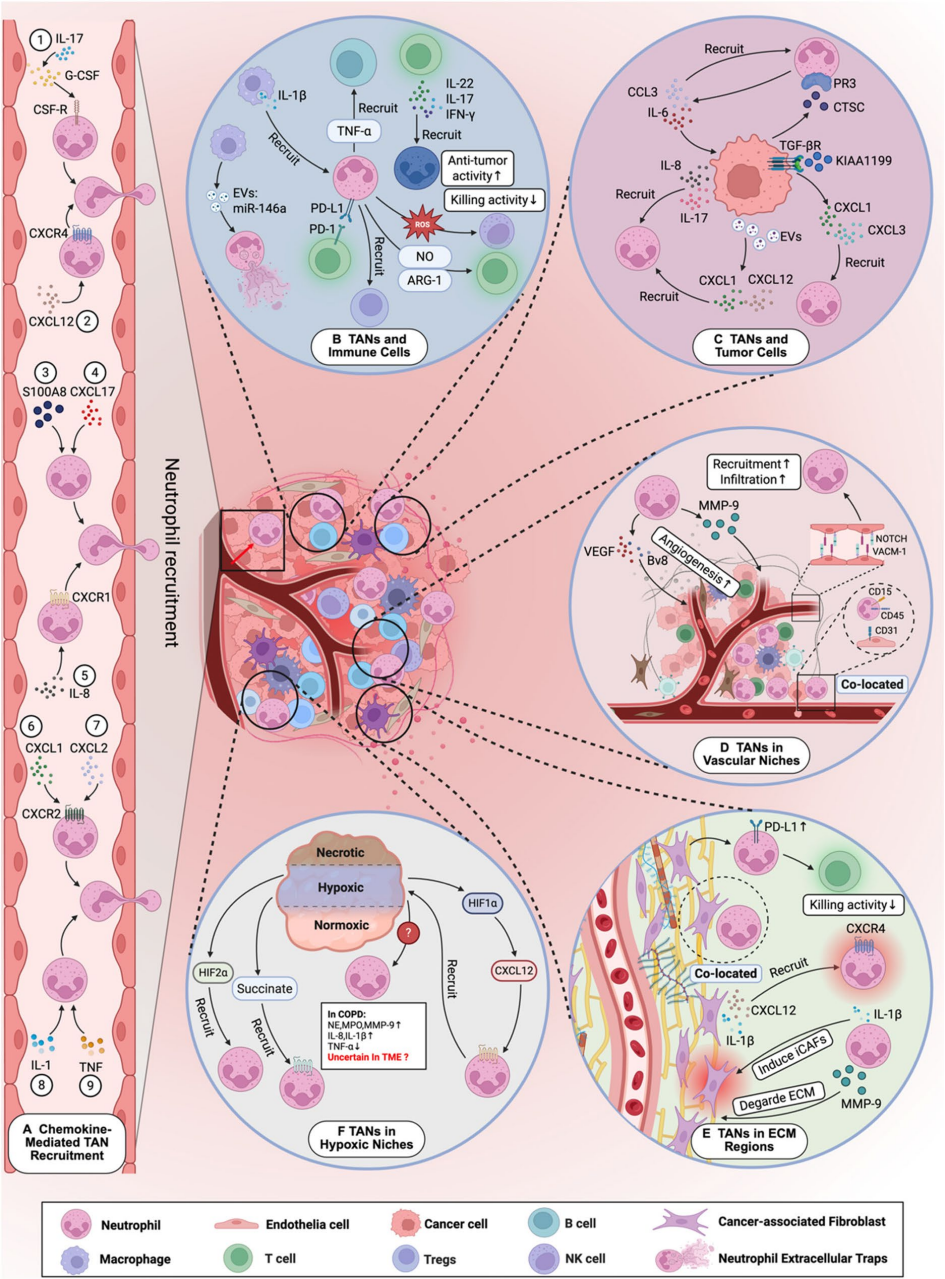
The factors influencing the spatiotemporal heterogeneity of tumor-associated neutrophils. This figure illustrates the multifactorial regulatory network that governs the spatial heterogeneity of tumor-associated neutrophils (TANs) within the tumor microenvironment (TME), integrating molecular cues, cellular interactions, and microenvironmental influences. Created in https://BioRender.com. (**A**) Chemokine-mediated recruitment of TANs. Tumor cells, stromal cells, and immune components secrete various chemokines (e.g., CXCL12, CXCL1/2/5) and cytokines (e.g., IL-8, G-CSF, IL-17 A), which bind directly or indirectly to cognate receptors on neutrophils. These interactions promote neutrophil extravasation and directional migration into the TME. (**B**) Crosstalk between TANs and immune cells. Macrophage-derived IL-1β and miR-146a-enriched exosomes enhance TAN recruitment and neutrophil extracellular traps (NETs) formation. TANs spatially inhibit T cell activity by engaging PD-L1/PD-1 interactions and suppress T cell activation via the secretion of arginase-1, reactive oxygen species (ROS), and nitric oxide (NO). Additionally, TANs attract regulatory T cells (Tregs) and B cells through TNF-α secretion, and impair NK cell cytotoxicity via ROS. Conversely, T cell-derived cytokines (IL-17, IL-22, IFN-γ) can recruit TANs that paradoxically enhance T cell activation. (**C**) TAN–tumor cell interactions. Tumor cells promote TAN recruitment through the secretion of IL-8, IL-17, and exosomes. The tumor cell-derived factor KIAA1199 upregulates the production of chemokines CXCL1 and CXCL3 by activating signaling pathways (e.g., TGF-β/SMAD3), thereby recruiting neutrophils. Cathepsin C, secreted by tumor cells, binds to membrane-bound proteinase 3 on TANs, promoting IL-6 and CCL3 release and further neutrophil recruitment. (**D**) TANs within vascular niches. CD15⁺CD45⁺ TANs preferentially localize to the perivascular niche. Endothelial cells, upon Notch activation, upregulate VCAM1 expression, facilitating TANs recruitment and infiltration. TANs in these regions promote angiogenesis by secreting VEGF, Bv8, and matrix metalloproteinase-9 (MMP-9). (**E**) TANs in ECM Regions. TANs exhibit spatial co-localization with cancer-associated fibroblasts (CAFs), which recruit TANs via the CXCL12/CXCR4 axis and IL-1β signaling. In turn, TANs promote the formation of inflammatory CAFs through IL-1β secretion and contribute to ECM degradation by releasing MMP-9, thereby facilitating tumor invasion. (**F**) TANs in Hypoxic Niches. Hypoxic regions within tumors upregulate HIF-1α and HIF-2α, promoting TAN accumulation. Tumor-derived succinate enhances TAN metabolic activity. Evidence from chronic obstructive pulmonary disease suggests that hypoxia-induced neutrophils exhibit elevated secretion of elastase, myeloperoxidase, lactoferrin, MMP-9, IL-8, and IL-1β, alongside reduced TNF-α production, a phenomenon yet to be fully characterized in cancer

The spatiotemporal heterogeneity of TANs is dynamically regulated by multiple signaling molecules within distinct ecological niches of the TME. These factors profoundly influence their phenotypic polarization, function, distribution, and interactions with the TME. Chemokines and inflammatory mediators within the TME mediate the recruitment and spatiotemporal positioning of neutrophils; epigenetic mechanisms also dominate the N1/N2 phenotypic polarization of TANs and the formation of NETs [60, 119]. Tumor-derived factors directly reprogram TAN phenotypes and induce NETs generation, while the hypoxic TME and ECM remodeling mediate neutrophil polarization through various signaling pathways, driving NETs-mediated metastatic dissemination. Furthermore, interactions between TANs/NETs and macrophages, T cells, etc., collectively promote tumor immune evasion (Fig. 1). Understanding these driving mechanisms provides a critical window for targeted intervention, offering the potential to reverse the pro-tumorigenic functions of TANs and enhance immunotherapy response, ultimately guiding the development of spatiotemporally precise therapeutic strategies.

### Genetic and molecular drivers

Within the TME, diverse chemokine signals activate intracellular pathways that orchestrate the organ-specific recruitment of neutrophils and promote their polarization toward immunosuppressive phenotypes. These chemokines also play a critical role in inducing NETs formation, thereby facilitating immune evasion (as summarized in Table 2). In parallel, the functional states and spatial localization of TANs are modulated by epigenetic reprogramming and tumor-intrinsic genetic alterations. Collectively, these regulatory inputs shape TAN recruitment, phenotypic polarization, and spatial distribution within the TME (Fig. 1).

**Table 2:** Roles of cytokines in neutrophil recruitment and modulation of the spatiotemporal landscape within the TME

**Cytokine**	**Receptors**			**Cancer**	**Role in tumour formation**		**References**
CXCL12	CXCR4			Multiple cancers	Drives TANs migration to CXCL12-high regions, promoting tumor progression.		[28, 35]
CXCL1	CXCR2			HCC, PDAC	Recruits CXCR2^+^ TANs, which are closer to the tumor cells, induces NETs formation, and facilitates tumor metastasis.		[26, 68]
CXCL2	CXCR2			Multiple cancers	Inhibits neutrophil apoptosis and maintains their survival, leading to neutrophil accumulation in tumor tissues.		[27, 39]
CXCL8	CXCR1,CXCR2			Multiple cancers	Recruits neutrophils into the TME, releases NETs, and suppresses T cell function.		[27, 120, 121]
IL-17	IL-17-CXCL1-CXCR2			Multiple cancers	Promotes neutrophil infiltration into into peritumoral stroma, releases NETs, and enhances tumor angiogenesis.		[5, 39, 48, 55, 122]
IL-8	CXCR1/2			Multiple cancers	Recruits neutrophils, inducing the expression of JAG2 in TANs, and promoting tumor immune evasion and increasing the extravasation potential of adjacent tumor cells.		[1, 3]
IL-1β	IL-1β-CXCL1-CXCR2			Multiple cancers	Recruits CXCR2 ^+^ TANs and triggers NETs formation, resulting in chemotherapy resistance.		[21, 123, 124]
GM-CSF	GM-CSF-JAK-STAT3			GC	Induces PD-L1 expression on neutrophils, activating them and conferring immunosuppressive functions.		[101]
TGF-β	TGFβ/SMAD3-CXCL1, CXCL3-CXCR2			CRC	Facilitates the aggregation of immunosuppressive neutrophils and drives tumor metastasis.		[55, 125]

Abbreviation: *TME* tumor microenvironment, *TAN* tumor-associated neutrophils, *HCC* hepatocellular carcinoma, *PDAC* pancreatic ductal adenocarcinoma, *NETs* neutrophil extracellular traps, *GC* gastric cancer, *CRC* colorectal cancer

#### Key signaling pathways

The dynamic recruitment and functional polarization of TANs are orchestrated by complex chemokine signaling networks within TME. Recent advances in spatial omics and single-cell sequencing technologies have unveiled the spatiotemporal heterogeneity and evolutionary dynamics of neutrophils across tumor compartments. As mentioned earlier, the intricate networks mediated by signaling pathways such as the CXCL12–CXCR4 and G‑CSF–CSF3R axes collectively determine the recruitment and functional states of neutrophils in different tumor regions, thereby constituting the core molecular basis of their spatiotemporal heterogeneity. A mechanistic dissection of chemokine–receptor signaling axes in the TME not only advances our understanding of neutrophil-mediated immune regulation but also informs the design of precision immunotherapies.

#### Key notes in NETs formation

The chemokine signaling axis also plays a central regulatory role in the induction of NETs. Cytokines such as CXCL1, CXCL8, IL-8, and G-CSF have been shown to induce NET formation by activating chemokine receptors on neutrophils [126, 127]. Among these, the G protein-coupled receptors CXCR1 and CXCR2 are essential mediators of NET release. This mechanism has been widely observed across multiple tumor types, highlighting its conserved and universal nature. In hepatocellular carcinoma and pancreatic ductal adenocarcinoma, elevated tumor-derived CXCL1 engages CXCR2 on the surface of neutrophils, leading to their recruitment and subsequent induction of NET formation [68, 128]. Mechanistically, this process involves activation of a canonical G protein-coupled receptor cascade: ligand binding triggers Gi subunit–dependent intracellular signaling, which ultimately leads to chromatin decondensation and the extracellular release of NETs [129]. Functionally, NETs facilitate tumor metastasis by forming extracellular chromatin structures that act as physical barriers, thereby obstructing immune cell attacks and enabling tumor immune evasion [129].

#### Epigenetic regulation and microenvironmental integration

In addition to extracellular signals, TAN behavior is modulated by intrinsic genetic and epigenetic programs that shape their phenotype and function in a context-dependent manner. Epigenetic reprogramming acts as a critical molecular mechanism that integrates environmental cues to modulate chromatin accessibility and transcriptional outputs in neutrophils.

At the molecular level, DNA methylation, histone modifications (e.g., acetylation, monoubiquitination), and chromatin remodeling collectively orchestrate TAN fate decisions. Spatially resolved single-cell multi-omics profiling has revealed that neutrophils within the TME probably undergo transcriptional and epigenetic reprogramming, leading to subtype differentiation, such as the terminally differentiated T3 subset [30]. Environmental signals in the TME induce context-specific chromatin remodeling, thereby regulating the accessibility of key transcriptional programs that drive immunosuppressive or pro-tumor phenotypes.

A compelling example of epigenetic–microenvironmental crosstalk is observed in hepatocellular carcinoma, where Deltex E3 ubiquitin ligase 2 facilitates CXCL2 and CXCL6 expression by modulating H2B K120 monoubiquitination, thereby enhancing TAN infiltration and establishing an immunosuppressive milieu through CD8⁺ T cell inhibition [130]. Similarly, the lineage-specific expression of histone deacetylase HDAC11 in neutrophils modulates cell migration and activation states through reshaping the epigenetic landscape of inflammation-related genes [131].

Hypoxia, a defining feature of the TME, is hypothesized to interact synergistically with epigenetic regulators to stabilize neutrophil plasticity. Although direct studies on TAN epigenetics under hypoxia are limited, available evidence suggests that hypoxia-inducible factors (HIFs) may form feedback loops with histone demethylases, influencing DNA methylation and histone modification patterns to sustain pro-tumor transcriptional programs [132]. While TANs’ short lifespan presents challenges for epigenetic research, these pathways nonetheless represent promising targets. Epigenetic drugs capable of altering transcriptional and metabolic programs may recondition the TME and restore neutrophil-mediated antitumor immunity.

#### Genetic factors and TAN phenotypic diversity

Tumor-intrinsic genetic mutations play a crucial role in shaping TAN phenotypes, influencing spatial distribution and functional behaviors. These mutations remodel the TME by modulating TAN recruitment patterns and functional polarization. Tumor-intrinsic mutations regulate chemokine production to recruit and activate TANs. For instance, KRAS-mutant tumors induce secretion of chemokines CXCL9/10 through activation of the RAS/MAPK pathway. MEK inhibitors can suppress chemokine production by inhibiting this pathway, thereby reducing TAN infiltration into tumors [52, 133]. In KRAS-driven lung adenocarcinoma models with SOX2 overexpression or NKX2-1 deletion, SOX2 directly mediates CXCL5 secretion to promote intratumoral TAN recruitment [134].

Genetic mutations also confer distinct spatial distribution characteristics to TANs. In PIK3CA-mutant lung squamous cell carcinoma, TANs are highly enriched in tumor cores, correlating with reduced vascularity, decreased intratumoral heterogeneity, and poorer prognosis [77]. TP53 mutation-mediated loss of EHF in tumors induces CXCL1 transcription, enhancing the recruitment of CXCR2^+^ neutrophils that exhibit closer spatial proximity to tumor cells. These CXCR2^+^ neutrophils can reduce the efficacy of immunotherapy and lead to drug resistance [26].

These findings systematically elucidate how tumor-intrinsic mutations regulate TAN recruitment, spatial organization, and functional reprogramming, providing a theoretical foundation for developing mutation-guided immunotherapy strategies. Combination therapies targeting mutation-associated pathways such as RAS/MAPK (e.g., MEK inhibitors) or CXCR2 signaling may enhance immunotherapy outcomes by modulating TAN spatial distribution and functional states [26, 133].

### Role of inflammatory

Inflammatory cytokines and chemokines are key mediators of the TME, profoundly shaping the recruitment, spatial localization, and functional polarization TANs. These mediators exert two primary effects: facilitating TAN infiltration into tumors, and modulating their phenotypic and functional heterogeneity (Fig. 1).

#### TAN recruitment and activation

##### Chemokines as chemoattractants 

Inflammation is a critical driver of tumor progression and therapeutic resistance [135]. Accumulating evidence suggests that inflammatory signals contribute significantly to the spatiotemporal heterogeneity of TANs. Chemokines are among the most prominent inflammatory mediators regulating neutrophil trafficking, facilitating TAN recruitment, a process observed throughout tumorigenesis, progression, and metastasis. Accordingly, targeting chemokine signaling may represent a promising strategy to prevent TAN infiltration and reshape the TME.

During tumor initiation, inflammatory stimuli induce local secretion of cytokines and chemokines that recruit neutrophils through receptor-specific pathways. Research indicates that tumor-derived chemokines CXCL1, CXCL2, and CXCL5 recruit CXCR2^+^ neutrophils and promote their accumulation [39]. Notably, TAN infiltration driven by cytokines is frequently associated with poor tumor prognosis. In hepatocellular carcinoma TME, CCL2^+^ or CCL17^+^ TANs demonstrate density correlations with tumor size, microvascular invasion, tumor encapsulation, differentiation grade, and staging [102, 136]. Furthermore, TP53 mutation–induced EHF deficiency enhances CXCL1 transcription and promotes CXCR2^+^ TAN-mediated chemoresistance and immune evasion [26]. These mechanisms highlight the essential role of inflammatory chemokines in guiding neutrophil migration into tumors. Upon recruitment, TANs are “educated” by the TME, acquiring tumor-promoting phenotypes that support invasion, metastasis, and immune suppression.

##### Enhanced vascular permeability and neutrophil extravasation

Beyond chemokine signaling, inflammatory cytokines also modulate vascular integrity to promote neutrophil extravasation. Tumor cells release mediators that increase endothelial permeability, facilitating neutrophil transmigration into tumor tissues. Circulating neutrophils undergo sequential steps of rolling, crawling, and transendothelial migration directed by chemokines and integrins. IL-1 and TNF-α facilitate neutrophil transendothelial migration, with IL-1 being particularly crucial for the initial anchoring step of neutrophil-endothelial interaction [137]. Notably, IL-1β, a master regulator of inflammation, not only enhances TAN infiltration but also contributes to the recruitment of immunosuppressive neutrophil subsets during anti-angiogenic therapy [123].

Mechanistically, both IL-1β and TNF-α enhance vascular permeability by disrupting endothelial tight junctions and adherens junctions. Similar phenomena were observed in a Pseudomonas aeruginosa pulmonary infection mouse model, where elevated IL-1β levels correlated with increased expression of CXCL-1, CXCL-2 chemokines, VCAM-1/ICAM-1 adhesion molecules, and CD11b^+^CD45^+^ cell recruitment. IL-1β-induced barrier disruption was significantly aggravated by co-treatment with TNF-α [124, 138]. Despite these findings, the contribution of other inflammatory mediators to neutrophil extravasation remains poorly defined and warrants further investigation. Elucidating these pathways is critical for developing interventions aimed at limiting TAN infiltration and overcoming tumor immune evasion.

#### Self-amplifying inflammatory feedback loops

The interaction between TANs and tumor cells can create positive feedback circuits that perpetuate inflammation and promote tumor progression. In lung cancer models, microbial colonization of the respiratory tract activates TANs, which secrete pro-inflammatory cytokines that stimulate lung-resident γδ T cells, amplifying local inflammation and facilitating tumor growth [139, 140]. Activated TANs further release chemokines and cytokines that recruit additional neutrophils and immune cells, establishing a self-reinforcing inflammatory niche. In clear cell renal cell carcinoma, epigenetic remodeling, including DNA demethylation and super-enhancer activation, leads to the transcriptional upregulation of inflammation-associated genes, driving TAN-dependent metastatic seeding in the lungs [141]. Moreover, tumor–bone axis interactions have been implicated in neutrophil mobilization: in lung cancer, osteocalcin⁺ osteoblasts in bone marrow remotely promote the recruitment of SiglecF^high^ TANs with pro-metastatic properties [142]. These findings collectively support the model wherein TANs not only respond to inflammatory signals but also propagate systemic pro-tumor inflammation.

Decoding the regulatory networks that govern TAN recruitment, polarization, and feedback amplification provides critical insight into the dynamic immunobiology of the TME. By targeting key inflammatory pathways, whether cytokine-mediated, microbiota-driven, or epigenetically reinforced, future therapies may reprogram neutrophil function to suppress rather than support tumor progression. Strategies aimed at interrupting self-sustaining inflammatory loops, promoting anti-tumorigenic like polarization, or leveraging microbial co-regulators represent compelling opportunities to complement and enhance current immunotherapeutic modalities.

### Immune factors

TANs occupy a central and multifaceted position in tumor immunology. Through dynamic and reciprocal interactions with tumor-associated macrophages (TAMs), dendritic cells (DCs), T cells, B cells, and natural killer (NK) cells, neutrophils actively modulate the immune landscape of the TME. These interactions are mediated by spatial co-localization, metabolic competition, and signaling crosstalk, collectively contributing to the establishment of immunosuppressive gradients, the induction of immune dysfunction, and the facilitation of immune evasion (Fig. 1). A comprehensive understanding of these multi-layered TAN–immune cell networks is essential for the rational design of combination immunotherapies targeting neutrophil-driven immune modulation in cancer.

#### Immune cell interactions: TANs as active nodes in immune regulation

##### Crosstalk with myeloid cells 

Recent studies have revealed that TANs, TAMs, and DCs form a complex spatial interaction network during tumor progression. In hepatic invasive tumors, TANs form stable spatial co-localization networks with Tregs and M2-polarized TAMs, particularly enriched in tumor invasion front regions [85]. These spatial assemblies shape immunosuppressive gradients and reinforce regional immune dysfunction.

Functionally, TAN–TAM interactions have been shown to co-activate the STAT3 signaling axis through oncostatin M and IL-11 secretion, driving tumor cell proliferation and invasiveness [33]. Notably, STAT3 acts as a pivotal signaling hub that not only directly regulates tumor cell behavior but also remodels the microenvironment through induction of immunosuppressive factors. Moreover, TAM-derived IL-1β regulates γδ T cell activation and supports pathological neutrophil accumulation [143].

Importantly, TANs function not solely as terminal effector cells but also as active regulators of the tumor immune microenvironment through the secretion of immunomodulatory cytokines and enzymes. Among these, TAN-derived CCL2 plays a reciprocal role in modulating macrophage recruitment and polarization, reinforcing the expansion of immunosuppressive myeloid networks [60]. Additionally, neutrophil-derived MPO profoundly impairs dendritic cell antigen presentation, particularly by inhibiting the activation of CD8⁺ T cells, through the induction of lipid peroxidation [144]. These findings delineate a complex spatial and functional interplay among myeloid subsets, wherein TANs act as central modulators of the immune landscape. This coordinated crosstalk contributes to the dynamic remodeling of the TME, progressively skewing its composition toward immune suppression and thereby facilitating tumor invasion and metastatic dissemination.

##### Bidirectional interactions with adaptive/innate immune cells

The interaction between TANs and T cells within the TME exhibits remarkable spatiotemporal heterogeneity and bidirectionality, playing a decisive role in shaping the balance between immune activation and suppression. On one hand, TANs can support T cell effector functions under specific immunological contexts. CD4⁺ and CD8⁺ T cells secrete IL-22, IL-17, and IFN-γ, which in turn induce colorectal cancer cells to produce CXCL1/2/3, thereby recruiting neutrophils that further enhance T cell activation [145]. In co-culture assays, HLA-DR⁺ TANs isolated from tumor tissues promoted TNF-α expression in CD3⁺ T cells, suggesting a capacity of TANs to amplify adaptive immunity under permissive spatial and cytokine conditions [31].

However, a growing body of evidence emphasizes the immunosuppressive functions of TANs, which appear to predominate in advanced or treatment-refractory settings. Spatial proximity between TANs and CD8⁺ T cells, particularly in the context of PD-L1 upregulation and diminished ROS production, has been associated with poor clinical outcomes in glioblastoma and other solid tumors [29, 58]. Close localization of PD-L1⁺ TANs and T cells correlates with T cell dysfunction and immune exclusion [29, 58]. Mechanistically, TAN-mediated suppression of T cell function operates through multiple, interrelated pathways. First, direct physical interactions between TANs and CD4⁺ T cells can impair T cell activation, proliferation, and viability [60, 146]. In parallel, metabolic reprogramming within TANs leads to the accumulation of immunosuppressive metabolites, including arginase-1, ROS, nitric oxide, and PGE₂ synthesized via FATP2-dependent arachidonic acid metabolism, which collectively inhibit T cell effector functions and promote an immunosuppressive microenvironment [110, 147, 148]. Furthermore, TAN-secreted metalloproteinases activate the TGF-β signaling pathway, reinforcing immune evasion while contributing to ECM remodeling and tissue restructuring [149]. Additionally, TANs secrete CCL2 and CCL17, which drives the recruitment of Tregs and TAMs, thereby consolidating localized immunosuppressive niches and further dampening anti-tumor immunity [102]. These immunoregulatory mechanisms reflect the ability of TANs to sculpt the immune landscape through both spatial positioning and effector function.

TANs also contribute to therapeutic resistance, particularly in the context of immune checkpoint blockade. In lung cancer, PD-1/PD-L1 inhibition induces tumor-derived CXCL5, which drives PD-L1⁺ TAN infiltration through the Paxillin–AKT signaling axis [150]. This cascade not only establishes self-reinforcing immunosuppressive circuits but also accelerates CD8⁺ T cell exhaustion, thereby attenuating the efficacy of checkpoint inhibitors [150]. These insights highlight a paradigm in which TANs serve as amplifiers of immune escape beyond conventional PD-1/PD-L1 interactions.

Beyond T cells, TANs engage in complex crosstalk with additional immune cell types. Through TNF-α, they recruit CD45⁺B220⁺CD138^−^ B cells, subsequently induce their differentiation into CD45⁻B220⁺CD138⁺ plasma cells, thereby promoting humoral immune responses [151]. Complement C3 signaling has been implicated in both TAN recruitment and NET formation, enhancing metastatic spread to the lungs [152]. Interactions with NK cells are particularly nuanced: while NK cells support the survival of sXBP1⁺ TANs, TANs suppress NK cytotoxicity via ROS release, forming reciprocal circuits that facilitate metastasis [103, 153].

Collectively, these findings underscore the multifunctional role of TANs as immune integrators, capable of either augmenting or suppressing anti-tumor responses in a spatially and temporally regulated manner. Deciphering the molecular networks that govern these TAN–immune cell interactions will be critical for identifying actionable targets. Therapeutic strategies aimed at disrupting TAN-driven immunosuppressive circuits or reprogramming their function toward immune activation may overcome current limitations in cancer immunotherapy and enhance durable clinical responses.

#### Immune evasion mechanisms and TAN spatial organization

##### Construction of immunosuppressive niches

Immune evasion in solid tumors is orchestrated through a multidimensional regulatory network, in which TANs function as central modulators of the immunosuppressive TME. This process involves both compositional reshaping of the immune milieu and the establishment of immunosuppressive barriers via metabolic reprogramming and coordinated signaling cascades, culminating in systemic attenuation of anti-tumor immunity.

Clinical evidence shows that in solid tumors such as gallbladder cancer liver invasion and hepatocellular carcinoma, TANs form a stable spatial co-localization pattern with Tregs and M2-type TAMs. This three-dimensional spatial arrangement gives rise to localized gradients of immunosuppressive cytokines and metabolic suppressors [85, 102]. Notably, the density of these multicellular aggregates correlates positively with tumor malignancy. Mechanistically, this spatial organization may be governed by CCL17/CCL2–CCR2/CCR4 chemotactic axes that mediate directional migration and coordinated positioning of immunosuppressive cells within the TME [102].

Mechanistically, tumor-derived cytokines such as CXCL5 can “educate” infiltrating neutrophils to acquire pro-tumor TAN phenotypes via activation of the PI3K/Akt and p38 MAPK signaling pathways. These reprogrammed TANs subsequently secrete CCL2 and CCL17, which further recruit F4/80⁺ TAMs and FoxP3⁺ Tregs, reinforcing a self-sustaining immunosuppressive circuit [25, 102]. In colorectal cancer, CXCL12/SDF-1–mediated neutrophil retention promotes persistent interaction between CD8⁺ T cells and CD15^high^ TANs, leading to aberrant GZMK secretion. GZMK, in turn, disrupts E-cadherin–mediated adhesion, facilitating tumor cell detachment and metastatic dissemination [154]. These findings delineate a spatially organized immune evasion system in which TANs coordinate with other suppressive immune subsets to construct localized immune-privileged niches. This architecture not only supports tumor progression but also provides a conceptual framework for combination immunotherapeutic strategies targeting neutrophil-mediated immune remodeling.

##### Immunoediting 

Immunoediting represents a dynamic process by which immune pressure selectively shapes the immunogenicity and cellular composition of the tumor. It proceeds through three interrelated phases: elimination, equilibrium, and escape. In the escape phase, tumor cells remodel the immune landscape through the secretion of immunosuppressive cytokines such as TGF-β, IL-6, and G-CSF, which collectively skew TAN polarization toward a pro-tumorigenic phenotype while suppressing anti-tumor subsets [155–157].

TGF-β, in particular, serves as a dual-function effector, promoting EMT and polarizing TANs, which contributes to CD8⁺ T cell suppression and immune dysfunction [25, 87, 149, 158]. In early-stage tumors, anti-tumorigenic TANs predominate and restrain tumor growth; however, as tumors evolve, pro-tumorigenic TANs become dominant, driving immune escape and metastatic progression [24, 25]. It underscores the role of immunoediting as a temporal driver of TAN functional plasticity. Through cytokine-mediated immune sculpting, tumors progressively reprogram TANs from anti-tumorigenic phenotypes in early disease toward immunosuppressive states in advanced stages, thereby facilitating immune escape, promoting metastasis, and reshaping the tumor–immune interface.

#### Impact of immunotherapy on TAN distribution and function

##### Immune checkpoint blockade

ICIs, particularly PD-1/PD-L1 and CTLA-4 antibodies, not only restore T cell function but also reshape the phenotype and spatial distribution of TANs. In a murine model of lung adenocarcinoma, ICIs induced the accumulation of IFN-responsive neutrophils within the TME. However, deletion of IRF1, a key IFN-responsive transcription factor in neutrophils, abrogated the therapeutic efficacy of checkpoint blockade, underscoring the necessity of neutrophil-intrinsic pathways for immunotherapy success [159]. Combination approaches targeting upstream inflammatory pathways have shown promise. In preclinical models of solid tumors and lymphomas, co-administration of the JAK inhibitor ruxolitinib with the anti–PD-1 antibody nivolumab significantly decreased the neutrophil-to-lymphocyte ratio and reduced myeloid suppressor cells abundance, while increasing the proportion of cytokine-producing T cells. Ruxolitinib also reversed T cell dysfunction, thereby augmenting the efficacy of immune checkpoint blockade [160].

##### Cytokine therapies 

Cytokine-based therapies, such as type I IFNs and IL-2, activate cytotoxic lymphocyte populations and modulate the spatiotemporal behavior of TANs. In an oral cancer model, knockdown of SET and MYND domain-containing protein 3 using antisense oligonucleotides sensitized tumors to anti–PD-1 therapy. scRNA-seq revealed enrichment of type I IFN response pathways in cancer cells, a shift toward effector/memory phenotypes in CD8⁺ T cells, and repolarization of TANs toward an anti-tumor state [161]. These findings suggest that combining epigenetic modulators or cytokine therapies with checkpoint blockade may yield durable immune reprogramming across multiple cell compartments.

Collectively, immunotherapy not only reinvigorates adaptive immune responses but also exerts a profound impact on the phenotypic plasticity and spatial organization of TANs. Therapeutic modulation of TAN behavior through immune checkpoint blockade, cytokine-based interventions, or combinatorial approaches involving JAK inhibition and epigenetic reprogramming emerges as a compelling strategy to enhance treatment efficacy and circumvent neutrophil-driven resistance within the TME.

### Influence of tumor-derived factors

Tumor cells actively secrete exosomes, soluble factors, and engage in direct cell-cell contact to precisely regulate the recruitment and phenotypic polarization of TANs. These factors not only drive the specific distribution of TANs and their transformation into a pro-tumorigenic phenotype but also induce NETs formation to promote the establishment of pre-metastatic niches. Furthermore, they orchestrate the immune evasion process within the TME through metabolic reprogramming and immunosuppressive signaling (Fig. 1). Understanding the complex interactions between tumor cells and TANs is crucial for developing more precise therapeutic approaches.

#### Tumor secretomes: drivers of TAN recruitment, polarization, and NET-mediated metastasis

The TME is shaped by a complex and dynamic interplay of tumor-derived exosomes, soluble factors, and post-translationally modified proteins, which collectively orchestrate the recruitment, spatial organization, and functional reprogramming of TANs. These tumor secretomes regulate both the physical positioning and phenotypic polarization of TANs, contributing to immune evasion, metastasis, and therapeutic resistance.

##### Modulation of TAN recruitment and polarization

Tumor cells secrete a diverse array of molecules that influence neutrophil chemotaxis and pro-tumorigenic transformation. In colorectal cancer, the secreted protein KIAA1199 interacts with the TGF-β receptor, activating the TGF-β/SMAD3 pathway and promoting CXCL1/3 production to recruit TANs [125]. Additionally, epithelial Notch1 signaling induces TGF-β release, facilitating TAN infiltration and enhancing metastatic potential [162].

Exosome-mediated mechanisms further contribute to TAN recruitment. In lung cancer, tumor-derived exosomes activate toll-like receptor 3 on pulmonary epithelial cells, triggering the secretion of CXCL1, CXCL2, CXCL5, and CXCL12, and promoting pre-metastatic niche formation [163]. These exosomes also increase endothelial permeability, thereby facilitating neutrophil transendothelial migration and tumor cell extravasation [164], such providing novel insights into neutrophil distribution across metastatic sites.

Soluble tumor-derived proteins also orchestrate neutrophil recruitment and establish a tumor-promoting TME, which is critical for tumor progression and metastasis. For example, breast cancer cells release cathepsin C, which activates membrane-bound proteinase 3 on neutrophils, leading to IL-1β maturation, NF-κB activation, and upregulation of IL-6 and CCL3, thereby attracting CD11b⁺Ly6G⁺ TANs to metastatic lung sites [122]. Similarly, pleiotrophin, produced by stromal components in breast cancer, recruits neutrophils via NF-κB–dependent pathways, fostering an immunosuppressive TME [165]. These findings underscore the complexity of tumor cell invasion/metastasis, presenting both challenges for targeted therapy and opportunities for precision intervention.

Additionally, post-translational modifications of tumor-derived proteins also shape neutrophil responses. In melanoma microenvironments, ubiquitinated X-linked inhibitor of apoptosis protein induces chemokine secretion through the TAB1/RIPK2 complex, facilitating TAN recruitment [166]. Beyond protein cues, noncoding RNAs and membrane communication influence TAN polarization. In pancreatic ductal adenocarcinoma, gap junction protein β3 facilitates cAMP transfer from tumor cells to neutrophils, enhancing survival and pro-tumorigenic skewing [167]. A circular RNA, Circ-PACRGL, derived from colorectal cancer, can increase TGF-β expression after absorbing miR-142-3P or miR-506-3P, leading to the polarization of neutrophils to the pro-tumorigenic phenotype and ultimately promoting tumor progression [168]. Through secreted proteins, exosomal signaling, post-translational modifications, and RNA-mediated communication, tumors spatially and phenotypically reprogram neutrophils to support immune suppression and metastatic progression. This complex regulatory network underscores the therapeutic potential of targeting tumor-derived cues to disrupt TAN-mediated tumor promotion.

##### Induction of NETs formation

Recent studies have revealed that TANs play a critical role in tumor metastasis through the release of NETs. NETs downregulate epithelial markers such as E-cadherin and Claudin-1, while upregulating mesenchymal markers including N-cadherin, Snail, and vimentin, thus facilitating EMT and tumor dissemination [113]. Tumor cells actively induce NETs formation via multiple molecular pathways. Key inducers include chemokines such as IL-8/CXCL8, growth factors like G-CSF, and cytokines such as TGF-β, all of which facilitate dynamic tumor cell dissemination [126, 127]. Moreover, epigenetic dysregulation markedly enhances the NETs-inducing capacity of tumor cells. For instance, KDM6A-deficient pancreatic cancer cells establish a microenvironment conducive to sustained neutrophil recruitment and NETs generation by upregulating CXCL1 expression [128].

NETs promote tumor dissemination through multiple complementary mechanisms. First, the proteolytic enzymes embedded within NETs disrupt endothelial junctions, increasing vascular permeability and facilitating tumor cell intravasation into the bloodstream, thereby promoting distant metastasis [169]. Second, postoperative stress responses in cancer patients induce a marked surge in circulating neutrophils and platelets. During this process, platelet activation is mediated via the TLR4–ERK5–GPIIb/IIIa signaling axis, enhancing their adhesion to and aggregation with circulating tumor cells. Concurrently, NETs released by activated neutrophils efficiently entrap these platelet–tumor cell aggregates [112]. This coordinated interaction substantially increases the migratory capacity of tumor cells, facilitating their retention within the pulmonary vasculature and promoting the formation of metastatic foci [112]. Moreover, in a microfluidics-integrated three-dimensional tumor–neutrophil co-culture model, NETs localized at the tumor–stroma interface were shown to promote collective invasion of the stroma by tumor cells, resulting in collective invasion [170]. Clinical data further validate these mechanistic insights, demonstrating that highly aggressive tumor cells possess enhanced NETs-inducing capacity, and that elevated NET deposition in metastatic niches correlates with poor patient outcomes [171, 172]. Collectively, these findings suggest that therapeutic strategies targeting key regulatory nodes of NETs formation, such as inhibition of CXCR1/2 signaling or enzymatic degradation of NETs, may provide effective approaches to disrupt pre-metastatic niche formation and limit metastatic progression.

#### Impact of tumor cells

Tumor cells dynamically shape the behavior and spatial localization of TANs through both soluble mediators and direct cellular interactions. These regulatory inputs not only coordinate TAN recruitment but also drive their phenotypic diversification across distinct tumor compartments, contributing to immune suppression, therapy resistance, and metastatic evolution.

##### Secreted factors

A growing body of evidence reveals that tumor-derived cytokines and chemokines orchestrate TAN recruitment and phenotypic reprogramming in a spatially selective manner. A systematic understanding of these secretory networks may reveal the molecular basis of TAN phenotypic plasticity and offer novel entry points for spatiotemporally informed therapeutic strategies.

In early stage of tumor development, tumor cells and microenvironmental components cooperatively secrete critical cytokines including G-CSF, GM-CSF, and IL-6, driving pathological neutrophil expansion [8]. This systemic regulation not only induces peripheral neutrophilia but also generates immature granulocyte subsets with distinct immunosuppressive phenotypes, establishing the foundation for subsequent TANs accumulation in local niches.

As tumors progress, chemokine-guided neutrophil recruitment becomes increasingly dominant. Multidisciplinary cancer studies reveal that tumor cells recruit receptor-specific neutrophils through direct secretion (e.g., IL-8) or indirect activation of stromal components such as platelet-derived CXCL5/7 chemokines [3, 4, 173, 174]. Notably, these chemotactic cues exhibit pronounced organ- and tumor-type specificity. Epithelial ovarian cancer and bladder cancer predominantly utilize the CXCR1/2-IL-8 axis [3, 174], whereas pancreatic ductal adenocarcinoma relies on IL-17 signaling to attract corresponding receptor-positive neutrophils [4]. Colorectal cancer cells uniquely produce IL-17, IL-33, TNF-α, and TGF-β to guide migration of IL-17R/ST2/TNF-αR/TGF-βR-positive neutrophils [4, 55, 162]. Such receptor expression heterogeneity highlights the capacity of distinct TME may shape specialized TANs subsets.

These phenotypically distinct populations subsequently localize within defined tumor niches, where they contribute to immune suppression, dampening of T cell cytotoxicity, and promotion of tumor progression and metastasis [3, 55, 122, 175]. Elucidating the spatiotemporal logic of tumor-derived secretory signals and their impact on TAN behavior will be crucial for advancing precision immunomodulation. Targeting the specific ligand–receptor pairs that mediate TAN recruitment and polarization may offer a promising strategy to inhibit neutrophil-driven immune escape and tumor progression.

##### Cell-cell interactions 

In the dynamic equilibrium of the TME, the biological behavior of TANs is regulated not only by soluble factors but also significantly influenced by physical contact with tumor cells, which markedly enhances their pro-tumorigenic functions.

Emerging research reveals temporally dependent signaling characteristics at the tumor-TAN contact interface. In a glioblastoma co-culture model, neutrophils initially display a pro-inflammatory, tumoricidal phenotype within the first 24 h. However, by 120 h, tumor cells induce functional tolerance in TANs through robust activation of the IL-6 and IL-1β signaling pathway, thereby shifting them toward a tumor-supportive state [176]. This spatiotemporal regulation suggests that tumor cells may functionally “reprogram” neutrophils through direct contact. Notably, the long non-coding RNA LINC01116 has been identified as a molecular bridge that enhances IL-1β expression by recruiting the DDX5 transcriptional regulatory complex to the IL-1β promoter region, thereby facilitating neutrophil recruitment [177]. The resulting TAN accumulation further supports tumor proliferation via cytokine-driven feedback loops.

Apoptotic tumor cells also play a key role in spatially structured neutrophil recruitment. By releasing IL-8, they attract TANs to apoptotic sites, where TANs interact with TAMs to promote M2 polarization and contribute to tumor recurrence, particularly in post-chemotherapy settings [178]. Notably, this signaling exhibits spatial specificity, with TANs preferentially clustering at apoptotic foci to form pro-regenerative immune niches.

Beyond local interactions, long-range inter-organ communication can also shape neutrophil behavior. For instance, lung adenocarcinoma cells engage in distant crosstalk with osteoblasts, mobilizing Siglec-F^high^ neutrophils that subsequently accumulate in the lungs and enhance metastatic progression [140, 142].

Despite these advances, the mechanisms governing direct tumor–TAN contact remain incompletely understood. Further elucidation of these contact-dependent signaling networks is essential to unravel how tumor cells spatially and temporally reprogram neutrophils. Such insights will be critical for defining new intervention points within the immune–tumor interface and for developing neutrophil-targeted strategies to disrupt pro-tumor crosstalk within the TME.

#### The role of tumor-secreted factors in modulating TAN behavior

After entering the TME, neutrophils undergo functional reprogramming into TANs, a transformation tightly regulated by tumor-derived factors. These factors promote TAN polarization toward immunosuppressive, pro-metastatic, and metabolically reconfigured phenotypes, thereby contributing to tumor immune escape, progression, and therapy resistance.

##### Immune suppression

Tumor cells exert precise control over TANs via secreted factor–receptor axes that modulate chemotaxis, phenotype, and gene expression. In hepatocellular carcinoma, stimulation of tumor cells with IL-18 induces phosphorylation of ACSL6 pS674, which subsequently activates the NF-κB signaling pathway. This activation promotes the transcription of neutrophil-attracting chemokines, including CXCL1 and CXCL5, thereby enhancing TAN recruitment. Once within the TME, TANs suppress CD8⁺ T cell cytotoxicity through the secretion of immunosuppressive mediators such as IL-10, arginase-1, and TGF-β, facilitating immune evasion and tumor progression [179].

In ovarian cancer, neutrophils undergo further phenotypic reprogramming upon tumor infiltration, differentiating into a distinct JAG2⁺ TAN subset. These TANs promote the differentiation of naïve CD4⁺ T cells into effector regulatory T cells via the JAG2–Notch1/RBPJ signaling pathway [57]. Notably, the spatial co-localization of JAG2⁺ TANs and effector regulatory T cells within the tumor further amplifies local immunosuppression, reinforcing the tolerogenic milieu [57].

Furthermore, tumor-derived exosomes contribute to the modulation of TAN function through post-transcriptional mechanisms. In particular, exosomal delivery of miR-362-5p downregulates CYLD, a known negative regulator of NF-κB signaling. The resulting sustained activation of NF-κB enhances neutrophil survival and immunosuppressive function [180]. These findings illustrate how tumor cells exploit TAN plasticity through both transcriptional and epigenetic mechanisms to construct a robust immunosuppressive microenvironment. Targeting these reprogramming pathways represents a compelling strategy to restore antitumor immunity and improve therapeutic outcomes.

##### Promotion of metastasis 

Certain tumor-secreted factors directly reprogram TANs from antitumor effectors into metastasis-promoting collaborators. In particular, tumor-derived cathepsin C activates proteinase 3 on neutrophils, promoting TAN recruitment and NETs formation to facilitate metastasis [122]. In colorectal cancer, tumor-derived CXCL2 gradients direct TAN homing to the tumor site, where TGF-β1 signaling induces expression of anterior gradient protein 2 (AGR2) in TANs. These AGR2⁺ TANs remodel the microenvironment to support tumor cell migration and are strongly associated with poor clinical prognosis [158]. In gastric cancer, tumor cell-secreted exosomes polarize TANs into the pro-tumorigenic subtype. Exosomes derived from pro-tumorigenic TANs transfer miR-4745-5p/3911 to gastric cancer cells, downregulating the expression of slit guidance ligand 2 and consequently enhancing cancer metastasis [181].

Interestingly, while microbial stimuli can transiently reprogram TANs to exert antitumor effects, neutrophil elastase and MMP-9 concurrently reshape tumor cell behavior. These proteases induce partial EMT in residual cancer cells, enabling migratory and invasive capabilities despite initial immune pressure [182]. Metabolite-driven inflammatory signaling also plays a critical role in shaping the metastatic potential of TANs. In nicotine exposure models, chronic STAT3 activation sustains neutrophil secretion of lipocalin-2, which promotes EMT and augments the pulmonary colonization capacity of tumor cells [183].

These mechanisms highlight the plastic and context-dependent nature of TANs in metastasis. Through both direct modulation of tumor cell phenotypes and remodeling of the pre-metastatic niche, TANs serve as critical mediators of metastatic progression. Understanding and disrupting these regulatory networks may offer new strategies to mitigate systemic dissemination and recurrence in cancer.

##### Alteration of TAN metabolism

The metabolic reprogramming of tumor cells produces a range of bioactive metabolites that profoundly alter the metabolic landscape of TANs, thereby reshaping their phenotype, functional output, and lifespan. This tumor–neutrophil metabolic axis has emerged as a key mechanism of immune evasion and metastatic progression.

One prominent contributor is the Warburg effect, wherein tumor cells preferentially engage in aerobic glycolysis, leading to excessive lactate accumulation and acidification of the TME [184]. This acidic milieu impairs the tumoricidal capacity of TANs and promotes their polarization toward an immunosuppressive pro-tumorigenic phenotype [185, 186]. Reprogrammed TANs subsequently secrete suppressive mediators that inhibit effective antitumor immune responses. In breast cancer, for example, tumor-recruited TANs undergo a glycolytic shift that disrupts pyruvate kinase M2 function and impairs STAT5 signaling, ultimately inducing T cell anergy [187]. Moreover, TANs themselves can modulate tumor cell metabolism through molecular cargo transfer. Neutrophils have been shown to deliver SPI1 mRNA to cancer cells via extracellular vesicles, enhancing SPI1 expression and upregulating glycolytic gene transcription. The resultant lactate production further promotes pro-tumorigenic TANs, establishing a metabolite-driven feedforward loop that reinforces tumor progression and immune suppression [186].

Collectively, these findings underscore the role of tumor-derived metabolites as potent modulators of TAN metabolism and immunosuppressive plasticity. Targeting this axis may yield novel therapeutic avenues, as inhibiting tumor-derived metabolic cues or reversing TAN metabolic reprogramming could restore neutrophil antitumor functions and synergize with existing immunotherapeutic strategies [188]. Understanding the intricate crosstalk between tumor metabolism and TAN functional states will be essential for developing precision interventions that rewire the immune–metabolic interface and enhance the efficacy of cancer immunotherapy.

### Vascular factors in TAN distribution and function

Tumor angiogenesis and the spatial distribution and functional activity of TANs are interconnected through a bidirectional regulatory axis (Fig. 1). Within the TME, the vasculature orchestrates TAN recruitment to perivascular regions via a range of chemotactic and endothelial-derived signaling cues. In turn, infiltrating TANs promote neovascularization by releasing pro-angiogenic mediators and matrix-degrading enzymes, such as MMP9, while concurrently compromising vascular integrity to enhance tumor cell intravasation and metastatic dissemination.

#### Angiogenesis as a spatial regulator of TAN localization and activity

The interplay between tumor angiogenesis and neutrophil infiltration represents a defining feature of the evolving TME, critically shaping the spatial distribution, activation states, and functional programs of TANs. These reciprocal interactions contribute not only to tumor progression and metastasis but also to resistance or sensitivity to therapeutic interventions.

The tumor vasculature serves as a specialized niche for TAN accumulation. In gliomas, CD15⁺CD45⁺ TANs are preferentially enriched in perivascular regions, suggesting functional crosstalk between neutrophils and endothelial cells [29]. This spatial co-localization suggests functional crosstalk between TANs and vascular endothelial cells, while their perivascular niche distribution implies potential involvement of neutrophils in tumor angiogenesis and vascular modulation. NETs also contribute to the angiogenic landscape. NETs have been shown to promote endothelial cell activation and neovascularization [73], and in turn, angiogenesis-driven recruitment of neutrophils establishes a positive feedback loop that sustains inflammation and vascular proliferation. Mechanistically, tumor cells engage Notch1 receptors on endothelial cells, leading to upregulation of VCAM1, which facilitates neutrophil adhesion and transmigration across the vascular wall [189].

Additionally, tumors can promote the intravascular retention of Ly6Clow neutrophils via CXCL1, leading to the formation of NET aggregates. These aggregates occlude blood vessels, consequently inducing downstream vascular hypoxia and necrosis. The resulting hypoxic TME around necrotic areas subsequently induces EMT in cancer cells through TGF-β signaling, ultimately facilitating metastasis. This study elucidates an active mechanism whereby neutrophils drive vascular occlusion, polymorphic necrosis, and metastasis through NET formation [80].

Together, tumor-driven angiogenesis and TAN infiltration constitute mutually reinforcing processes. Their spatial and molecular coordination not only facilitates immune evasion and tumor expansion but also promotes metastatic dissemination by enabling neutrophil trafficking into peripheral tissues and supporting pre-metastatic niche formation. Disrupting this neutrophil–vascular axis may offer a promising therapeutic strategy to simultaneously impair tumor vascularization, suppress TAN-mediated immunosuppression, and enhance the efficacy of anti-angiogenic and immunomodulatory therapies.

#### How TANs promote or inhibit tumor vascularization

While tumor vascularization plays a pivotal role in guiding the spatial distribution of TANs, accumulating evidence indicates that TANs actively contribute to tumor angiogenesis, thereby creating a reciprocal amplification loop between neutrophil infiltration and vascular remodeling.

Clinical and preclinical studies have demonstrated that TAN density correlates positively with microvessel density in multiple tumor types, including high-grade myxofibrosarcoma and hepatocellular carcinoma [5, 190]. Additionally, TANs promote tumor angiogenesis through a range of complementary pathways. First, neutrophils secrete Bv8 and VEGF, two potent pro-angiogenic factors that directly stimulate endothelial cell proliferation and neovascularization [30, 191, 192]. In glioblastoma, VEGFA⁺ TANs not only enhance angiogenesis but also exert pronounced immunosuppressive effects through crosstalk with the tumor stroma [193]. Second, neutrophil-derived MMP-9 facilitates angiogenic switching by mobilizing ECM-bound VEGF-α and enabling its interaction with VEGFR2 on endothelial cells, as shown in the RIP1-Tag2 mouse model of pancreatic neuroendocrine tumors [40, 194]. Additionally, TANs release oncostatin M, which activates the JAK–STAT signaling pathway in tumor cells, leading to upregulation of VEGF expression [195]. Tumor-secreted G-CSF further enhances TAN-mediated angiogenesis by inducing PROK2 expression [196, 197]. Finally, complement component C5a plays a dual role by facilitating TAN recruitment and triggering activation upon vascular endothelial contact. The formation of the membrane attack complex induces TANs to release NETs, which increase vascular permeability and promote melanoma cell intravasation and systemic dissemination [198]. These molecular and spatially coordinated pathways underscore the multifactorial role of TANs in promoting tumor vascularization, with implications for both local tumor expansion and distant metastatic spread.

Importantly, the therapeutic relevance of this axis is gaining recognition. Targeting TAN-specific pathways, particularly in combination with existing immunotherapies or anti-angiogenic agents, may offer a novel strategy to overcome neutrophil-driven resistance and improve clinical outcomes in solid tumors.

### Extracellular matrix

The ECM is a fundamental component of the TME, exerting profound influence on the spatial distribution, functional behavior, and phenotypic plasticity of TANs. TAN–ECM interactions contribute to tumor progression, angiogenesis, and immune modulation through a variety of biophysical and biochemical mechanisms (Fig. 1).

#### ECM components and TAN recruitment

##### Chemotactic signaling and CAF-mediated modulation

Dynamic remodeling of the ECM is a hallmark of cancer progression, driven by excessive collagen deposition and matrix cross-linking primarily mediated by cancer cells and CAFs. The resulting fibrotic matrix not only forms a physical barrier that restricts immune cell infiltration but also shapes a chemotactically active microenvironment that governs TAN localization and behavior [199–201]. Emerging studies suggest that the ECM functions far beyond passive structural support; rather, it serves as an active biochemical signaling platform that regulates TAN recruitment in both time- and space-dependent manners. Among ECM-regulating cells, CAFs act as central coordinators of neutrophil positioning and activation via bidirectional interactions.

Clinical observations demonstrate significant spatial colocalization of TANs and CAFs in melanoma and pancreatic ductal adenocarcinoma, suggesting potential functional crosstalk between these cellular compartments [46]. CAFs recruit neutrophils via multiple signaling pathways, including the SDF1a/CXCR4 axis [202], IL-1β secretion [203], and activation of CXCL8 and CSF1-R signaling pathways [120]. In renal cell carcinoma, CXCL1–CXCR2-mediated CAF activation triggered by renal tubular epithelial injury fosters neutrophil infiltration, fueling pro-tumor inflammation, angiogenesis, and even contralateral metastasis [204]. Notably, CAFs enhance tumor invasion and metastasis by promoting NETs in TANs. Comparative studies across murine models have demonstrated that CAF-induced NETs is significantly enhanced compared to normal fibroblasts, both in vitro and in vivo [46]. CAF-secreted amyloid β has been identified as a key molecular driver of this effect. Conversely, TAN-derived IL-1β reinforces stromal inflammation by inducing inflammatory CAF polarization and activating IL-6/STAT3 signaling in tumor cells via paracrine feedback loops [205]. This reciprocal interaction contributes to a pro-tumorigenic and immunosuppressive niche.

##### Matrix metalloproteinases (MMPs) 

TANs are a major source of MMPs, a family of extracellular proteolytic enzymes that mediate ECM degradation, endothelial permeability, and growth factor release. Among them, MMP-9 is the most abundantly secreted neutrophil-derived protease and plays a central role in tumor angiogenesis. During cancer progression, MMP-9 secreted by TANs degrades the ECM and stimulates angiogenesis in endothelial cells [194]. In addition to MMP-9, tissue inhibitor of metalloproteinase-1 (TIMP-1), a natural MMP inhibitor, has demonstrated complex regulatory effects on neutrophil-mediated tumor dynamics. A clinical study in patients with stage I–III primary breast cancer reported elevated expression of TIMP-1 in tumor-infiltrating neutrophils. Paradoxically, increased TIMP-1 expression was associated with enhanced tumor cell motility, potentially through the induction of EMT, thus reflecting a context-dependent role of the MMP–TIMP axis in neutrophil-driven metastasis [206]. Consistently, high TIMP-1 expression has been broadly associated with poor prognosis across multiple tumor types [207–209].

However, contrasting evidence suggests that it is the imbalance, rather than the absolute levels, of MMPs and TIMPs that may determine clinical outcomes. A study examining the prognostic implications of MMP-9 and TIMP-1 co-expression revealed that a high MMP-9/TIMP-1 ratio is more reliably linked to adverse outcomes, as this enzymatic disequilibrium facilitates unchecked ECM degradation, tumor cell invasion, and metastatic dissemination [210]. These findings underscore the complexity of the TME and highlight the importance of considering both spatial context and molecular ratios when evaluating the functional roles of MMPs and their inhibitors in TAN-mediated tumor progression.

Together, these findings position the ECM and its cellular regulators such as CAFs as key orchestrators of TAN spatial behavior and immune function [121]. By modulating ECM remodeling, signaling gradients, and NETs induction, tumors exploit ECM–TAN interactions to promote angiogenesis, invasion, and immune evasion. Targeted disruption of ECM–neutrophil crosstalk holds promise for enhancing the efficacy of immunotherapeutic interventions and restraining metastatic progression.

#### ECM remodeling by TANs

Through the secretion of proteolytic enzymes and ROS, TANs actively remodel the ECM. This remodeling process not only disrupts the structural integrity of the ECM but also generates permissive tracks for tumor cell migration, thereby facilitating local invasion and metastatic dissemination.

##### Secretion of proteases

TANs contribute to tumor progression in part through the secretion of extracellular proteases, including neutrophil elastase, cathepsin G, and MMPs, that orchestrate dynamic remodeling of the ECM. This proteolytic activity facilitates tumor cell invasion and metastasis by degrading structural ECM barriers and generating migratory conduits for tumor dissemination.

In osteosarcoma, for instance, neutrophils and tumor-associated stromal cells cooperatively activate fibroblasts, inducing ECM remodeling and establishing anchoring sites that support tumor cell attachment and migration, thereby accelerating metastatic spread [211].Similarly, hepatic accumulation of inflammatory FSCN1⁺ macrophages and HERC6⁺ neutrophils has been implicated in promoting liver fibrosis, a process likely mediated through the CCR2/STAT1/NF-κB/ERK signaling cascade, which also modulates ECM integrity and inflammation [212].

Neutrophil-driven ECM proteolysis is primarily mediated by a repertoire of serine proteases, most notably MMPs, neutrophil elastase, and cathepsin G. These enzymes reshape the TME, influence cancer cell signaling, and contribute to the establishment of pre-metastatic niches. Neutrophil elastase, in particular, has emerged as a key effector of ECM remodeling. Through ECM degradation, it alters cell–matrix interactions and activates downstream signaling pathways that support tumor proliferation and invasion [213]. Collectively, these findings highlight the critical role of TAN-derived proteolytic enzymes in ECM remodeling and underscore their significance in modulating tumor cell dynamics, stromal interactions, and metastatic potential.

##### Release of reactive oxygen species (ROS) 

ROS can induce oxidative modifications of ECM components, thereby facilitating tissue remodeling, enhancing tumor cell migration, and supporting metastatic colonization.

In breast cancer models, loss of the mitochondrial transmembrane protein TMEM126A induces ROS production and mitochondrial membrane depolarization, which promotes ECM remodeling and EMT. The ROS scavenger reversed the TMEM126A-mediated ECM remodeling and EMT [214]. Multiple studies have demonstrated that TANs can secrete ROS [122, 153]. However, the relationship between ROS secreted by TANs and ECM remodeling has not been fully elucidated.

ROS also play a critical role in regulating neutrophil NETs formation. CAFs have been identified as potent inducers of suicidal NETs via a ROS-dependent mechanism. Specifically, CAFs secrete amyloid β, which binds to CD11b receptors on neutrophils and activates ROS production, inducing the formation of NETs. The resulting NETs contribute to tumor growth both locally and systemically by fostering a pro-inflammatory and immunosuppressive TME [46].

Together, these findings underscore the pivotal role of ROS in neutrophil-driven ECM remodeling, NETs formation, and stromal reprogramming. Therapeutic strategies aimed at disrupting these ROS-mediated pathways may hold promise for attenuating TAN- and CAF-mediated tumor progression while preserving neutrophil-dependent host defense.

#### Influence of ECM on TAN function

The ECM exerts a profound influence on the phenotype, survival, and functional activity of TANs, thereby modulating the immune landscape of the TME.

##### Phenotypic modulation

The biochemical composition and mechanical properties of the ECM, particularly its stiffness and density, have been shown to shape TAN polarization. A rigid ECM enriched in fibronectin and collagen fibers can induce a shift toward a pro-tumorigenic phenotype, enhancing TAN-mediated immunosuppression and tumor support. Notably, matrix stiffness has been demonstrated to facilitate neutrophil NETs formation via fibronectin-dependent pathways. In vitro models revealed that fibronectin significantly amplifies neutrophil activation, promoting excessive NETs production and upregulating the release of pro-inflammatory cytokines and chemokines [215]. These effects suggest a critical role for ECM mechanics in neutrophil reprogramming.

##### Survival and activation

ECM components also provide essential survival signals that extend the lifespan and sustain the activity of TANs within the TME. The bidirectional crosstalk between stromal fibroblasts and immune cells reinforces ECM remodeling and immune suppression. CAFs secrete stromal cell-derived factor-1, which facilitates the recruitment of monocytes into the TME [202]. They also release IL-4, IL-13, and CXCL14, which promote the polarization of macrophages and neutrophils toward immunosuppressive pro-tumorigenic TAMs and TANs, respectively [216]. Reciprocally, TANs and TAMs secrete profibrotic mediators such as TGF-β, further stimulating CAF activation and ECM deposition.

##### Feedback loops and pre-metastatic conditioning

This immunofibrotic feedback loop is further amplified by tumor-derived soluble factors including TGF-β, platelet-derived growth factor, and ROS, which drive CAF differentiation and ECM remodeling [217]. In the context of colorectal cancer liver metastasis, fibroblast growth factor 19 has been identified as a key regulator. Fibroblast growth factor 19 promotes the polarization of hepatic stellate cells into inflammatory CAFs via FGFR4/JAK2/STAT3 signaling. These inflammatory CAFs, in turn, facilitate CRC cell colonization in the liver through the secretion of complement component C5a and IL-1β, thereby promoting neutrophil recruitment and NET formation [218].

Collectively, these findings underscore the ECM’s dual role as both a structural scaffold and a dynamic modulator of neutrophil phenotype and function. Targeting ECM–TAN interactions may represent a novel therapeutic avenue for disrupting pro-tumorigenic signaling networks and reconditioning the immunosuppressive TME.

#### ECM and immune evasion

The remodeled ECM plays a pivotal role in facilitating immune evasion within the TME, serving not only as a structural barrier but also as an active immunomodulatory interface. TANs, through their proteolytic remodeling of the ECM, contribute to the formation of fibrotic niches that physically and biochemically exclude effector immune cells, thereby creating immune-privileged zones favorable for tumor progression.

##### Physical barrier to immune cell infiltration

One of the primary mechanisms by which the ECM mediates immune evasion is through its mechanical properties. A dense and stiff ECM impedes the infiltration of cytotoxic T lymphocytes and NK cells into tumor parenchyma. TANs contribute to this exclusionary architecture by depositing and modifying matrix components, thereby enhancing tissue rigidity. Mechanotransduction pathways, such as the Rho/ROCK axis, are activated in T cells encountering stiff fibrotic matrices, which dampens their motility and cytotoxic functions [219].

Type I collagen, a key component of the fibrotic ECM, has been implicated in establishing an immunosuppressive milieu in various malignancies, including hepatocellular carcinoma. Cirrhotic ECM enriched in type I collagen activates the DDR1–NFκB–CXCL8 signaling cascade, which promotes NETs formation and shields tumor cells from T-cell-mediated killing [220]. These NETs not only form a physical barrier but also produce immunosuppressive molecules that further impair T cell function.

CAFs also play a central role in mediating immune suppression through their interactions with TANs. The interaction between the S100A12^+^ neutrophil subset and CAFs upregulates ECM-related genes, forming a dense physical barrier that suppresses tumor immunity [221]. Furthermore, neutrophil-derived extracellular DNA can stimulate pancreatic stellate cells to generate a dense fibrotic stroma. This creates a reinforcing feedback loop between TANs and stromal fibroblasts, facilitating tumor proliferation. Notably, deletion of the receptor for advanced glycation end products in pancreatic stellate cells abrogates this fibrogenic response and mitigates tumor progression [222].

Despite mounting evidence implicating TANs in ECM remodeling and immune evasion, the precise molecular mechanisms governing their bidirectional interactions remain incompletely defined. This knowledge gap is partly due to the inherent technical difficulty of isolating and preserving TANs, which are short-lived and sensitive to ex vivo manipulation. Nevertheless, given their functional significance in shaping the immunosuppressive stroma, TAN–ECM interactions warrant deeper investigation. Emerging spatial transcriptomic and high-resolution imaging platforms hold promise for resolving the spatial crosstalk between TANs and ECM components at single-cell resolution. A more granular understanding of these interactions could unlock novel therapeutic targets aimed at disrupting the pro-tumor matrix barrier and restoring immune surveillance. Strategies that selectively target TAN-mediated ECM remodeling, without compromising neutrophil host defense functions, may synergize with checkpoint blockade and other immunotherapies to improve clinical outcomes in solid tumors.

### Hypoxia

Hypoxia, a defining feature of the spatiotemporal heterogeneity within the TME, profoundly influences the recruitment, polarization, and functional plasticity of TANs. The crosstalk between TANs and hypoxic signals orchestrates multiple pro-tumorigenic processes, including immune evasion, ECM remodeling, neovascularization, and resistance to therapy (Fig. 1).

#### Hypoxia-induced recruitment of TANs

Recent spatial transcriptomic analyses in glioblastoma have revealed that neutrophils are preferentially enriched within hypoxic tumor niches, and the presence of these hypoxia-associated immune clusters correlates with poor clinical outcomes [223]. This spatial convergence suggests that neutrophils may facilitate tumor progression through hypoxia-dependent mechanisms, although the precise molecular programs and anatomical localization of these TAN populations remain to be fully elucidated.

##### Chemokine-mediated recruitment

Hypoxia within the TME exerts a pivotal influence on the spatial organization of TANs. Recent studies have identified that dcTRAIL-R1⁺ neutrophils, which possess vascular remodeling potential, are preferentially enriched in tumor regions characterized by oxygen deprivation and elevated glycolytic activity. These neutrophils express high levels of VEGF-α and contribute to vascular remodeling within hypoxic tumor cores [30]. This specific localization pattern is tightly linked to chemokine-mediated recruitment mechanisms. In gliomas, HIF-1α markedly upregulates the expression of CXCL12, a potent chemotactic factor that guides neutrophil migration toward hypoxic niches within the tumor [54, 55]. This oxygen-dependent chemotactic axis underlies the characteristic spatial distribution of TANs in solid tumors and presents potential opportunities for the development of hypoxia-targeted immunomodulatory strategies.

##### Role of hypoxia-inducible factors (HIFs)

HIFs, key transcriptional regulators activated under low oxygen conditions, are critically involved in promoting tumor cell proliferation, migration, invasion, and angiogenesis. These factors also modulate therapeutic responsiveness across cancer types [52, 224, 225]. Beyond oncology, HIFs play pivotal roles in other hypoxia-driven pathologies. For instance, in myocardial infarction, localized ischemic hypoxia activates HIFs that play pivotal roles in cellular functions. Research indicates that HIF-2α regulates the expression of survivin (Birc2) by binding to hypoxia response elements in its promoter region, thereby enhancing neutrophil infiltration in ischemic tissues [226]. Despite extensive investigation into HIF signaling in TAMs and T cells, their roles in TAN biology remain relatively underexplored [225].

Notably, emerging evidence indicates that HIF-1α is a central mediator of NETs formation under hypoxic conditions. Elevated levels of HIF-1α in neutrophils facilitate the release of NETs, which contribute to tumor cell trapping, immune evasion, and metastasis [227, 228]. Downregulation of HIF-1α expression inhibits tumor metastasis and is associated with prolonged survival in preclinical models [225, 227]. Collectively, these findings underscore the significance of HIFs in TAN-mediated tumor progression and highlight HIF-1α as a promising therapeutic target. Further elucidation of HIF-regulated pathways in TANs may enable the development of novel interventions aimed at disrupting the hypoxia–neutrophil axis to improve long-term clinical outcomes.

#### Functional modulation of TANs by hypoxia

##### Induction of pro-tumorigenic phenotypes

Hypoxia plays a pivotal role in shaping the phenotypic plasticity of TANs and modulating their interactions with tumor cells. Experimental evidence indicates that relief of intratumoral hypoxia reduces TAN recruitment, while neutrophils in the TME exhibit enhanced anti-tumor activity. This effect is mediated by the upregulation of NADPH oxidase-derived ROS and MMP-9, both of which contribute to cytotoxic and matrix-degrading functions [229]. Comparable proinflammatory responses have been observed in the context of alpha-1 antitrypsin deficiency, where neutrophils under hypoxic stress exhibit elevated secretion of neutrophil elastase, MPO, lactoferrin, and MMP-9. These changes are accompanied by increased production of IL-8 and IL-1β, while the expression of TNF-α is diminished [230]. Collectively, these findings suggest that hypoxia not only enhances neutrophil inflammatory potential but also fine-tunes their functional output in a context-dependent manner, potentially shifting TANs toward pro-tumorigenic or anti-tumorigenic roles depending on oxygen availability and the surrounding immunometabolic landscape.

##### Promotion of NETs formation

Mitochondrial metabolism within the tumor intensifies tumor hypoxia, which in turn promotes the accumulation of neutrophils and induces the formation of NETs. This process is driven by increased CXCL5 secretion and upregulation of HIF-1α expression, which together amplify NETs production in the hypoxic TME [231]. In turn, NETs further stimulate mitochondrial activity within tumor cells, aggravating local hypoxia and establishing a self-reinforcing pro-metastatic loop. Functionally, NETs released by TANs capture circulating tumor cells and facilitate their extravasation and colonization. Elevated HIF-1α not only promotes NETs formation but also enhances tumor cell migratory capacity, invasiveness, and acquisition of stem-like traits, all of which are critical for metastatic progression [227]. As such, HIF-1α represents a compelling therapeutic target for metastasis prevention.

#### Hypoxia and immune suppression

Hypoxia plays a critical role in shaping the immunosuppressive landscape of the TME, particularly through the functional reprogramming of neutrophils. In CD71⁺ neutrophils, hypoxic stress activates glycolytic metabolism, resulting in elevated lactate production. This metabolic shift promotes histone lactylation, which in turn upregulates the expression of ARG1. ARG1 depletes extracellular arginine, a key nutrient for T cell proliferation and effector function, thereby suppressing T cell responses and facilitating tumor immune escape [232]. The immunosuppressive role of hypoxia-activated neutrophils is further illustrated in inflammatory disease models. In cutaneous leishmaniasis, for example, neutrophil accumulation intensifies local hypoxia through excessive ROS production. This process enhances the expression of granzyme B in CD8⁺ T cells, reflecting a hypoxia-driven reshaping of T cell cytotoxic programs within inflamed tissues [233]. These findings collectively suggest that neutrophils under hypoxic conditions contribute to a self-reinforcing cycle of inflammation and immune suppression, with parallels in the tumor context.

#### Hypoxia and therapeutic resistance

Hypoxia plays a critical role in enhancing the persistence and immunosuppressive potential of TANs. A key mechanism involves the stabilization of HIF-1α, whose degradation is inhibited under oxygen-deprived conditions [234]. Additionally, Hif-2α promotes neutrophil survival by regulating the expression of the anti-apoptotic factor cellular inhibitor of apoptosis protein-1 [226].

Moreover, transcriptomic profiling has shown that hypoxia-associated gene signatures are enriched in pro-tumorigenic TANs, which exhibit elevated NET formation and increased expression of immune checkpoint molecules. These features contribute to the creation of an immunosuppressive TME that dampens adaptive immune responses. In contrast, therapeutic reversal of hypoxia, such as through microwave thermochemotherapy, can reactivate TAN cytotoxicity. Specifically, reoxygenation leads to the induction of MNDA⁺ TANs, a subset with enhanced tumoricidal capacity, highlighting the functional plasticity of TANs in response to oxygen tension. Mechanistically, hypoxia has also been shown to amplify inflammatory signaling via IL-1β, thereby promoting metastatic dissemination [235].

The presence of hypoxia-conditioned TANs within distinct tumor niches presents a formidable barrier to the efficacy of cancer therapies. These TANs contribute to spatially organized immune evasion by reinforcing localized immunosuppressive circuits and impairing the recruitment and activation of effector lymphocytes. As a result, the therapeutic response to ICIs and other immunomodulatory agents is often attenuated in hypoxic tumors [236]. Understanding how spatially and temporally restricted hypoxia shapes TAN fate and function is critical for the rational development of combinatorial treatment strategies. Strategies aimed at disrupting hypoxia-regulated TAN signaling circuits, either by targeting HIF-dependent transcriptional programs or by reprogramming TANs toward antitumor phenotypes under hypoxic stress, may restore immune competence, overcome therapeutic resistance, and improve clinical outcomes.

### Role of NETs in the TME

**Fig. 2:**
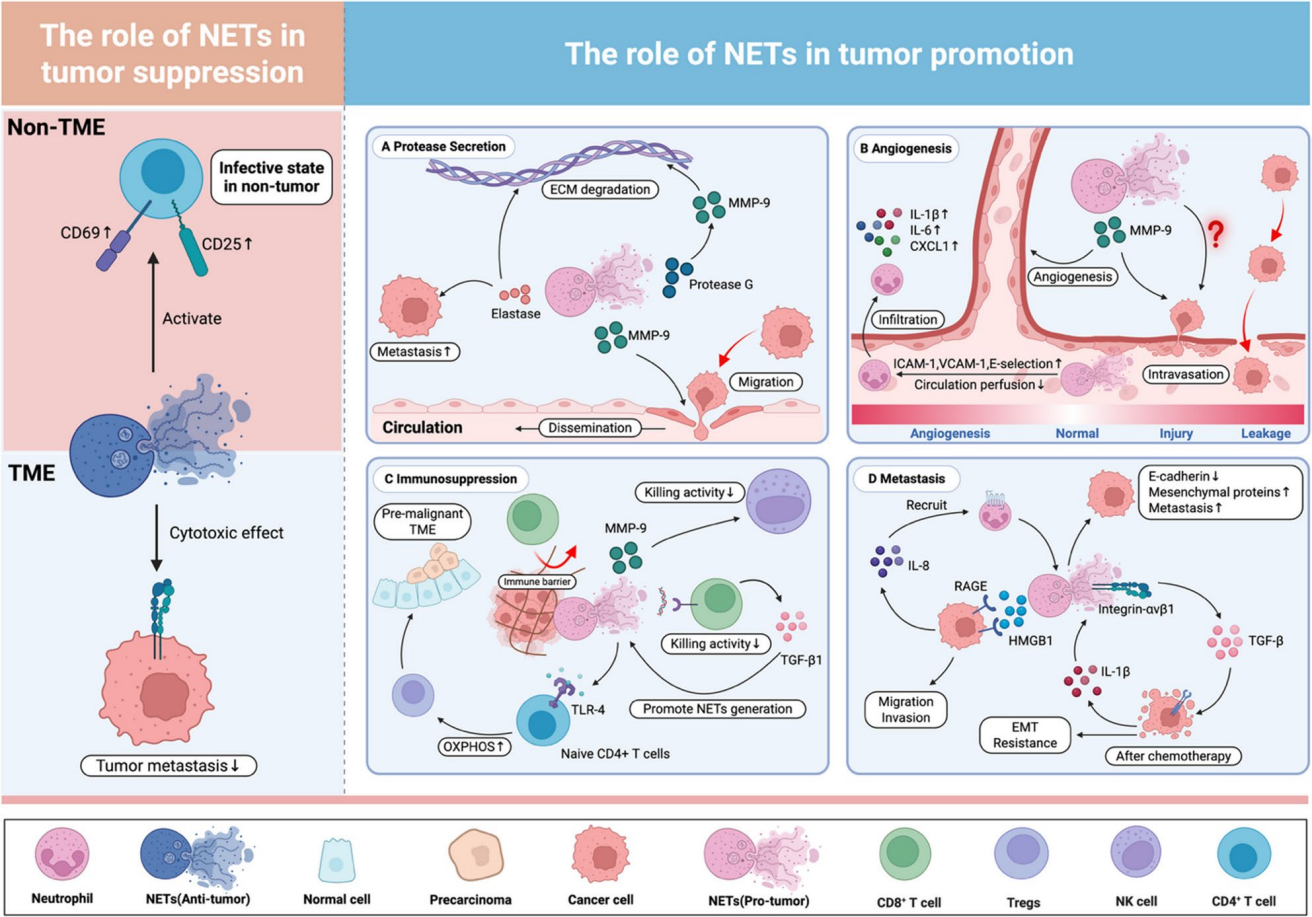
Dual roles of neutrophil extracellular traps (NETs) in tumor progression. This figure depicts the dual and context-dependent roles of NETs within the TME, highlighting both their anti-tumorigenic and pro-tumorigenic effects. Created in https://BioRender.com. (I) Anti-tumorigenic functions of NETs. NETs can immobilize tumor cells through integrin-mediated adhesion, thereby inhibiting migration and inducing necrotic cell death. In infectious settings, NETs enhance T cell immunity by upregulating activation markers CD25 and CD69 on CD4⁺ T cells, promoting ZAP70 phosphorylation, lowering the activation threshold, and augmenting T cell responsiveness. (II) Pro-tumorigenic functions of NETs. (**A**) Promotion of tumor invasion via proteolytic activity. NETs secrete neutrophil elastase (NE), which degrades the ECM to facilitates tumor metastasis. In addition, NETs release MMP-9 either directly or via neutrophil activation, further contributing to ECM degradation, disruption of endothelial barrier integrity, and promotion of tumor cell invasion and intravasation. Together, these proteolytic enzymes mediate ECM remodeling and indirectly enhance metastatic dissemination. (**B**) Regulation of tumor angiogenesis. NETs exert multifaceted effects on the tumor vasculature. NETs secrete MMP9 to induce endothelial cell apoptosis, enhance vascular permeability, and induce vascular leakage, providing conduits for cancer cell dissemination. In parallel, MMP-9 facilitates angiogenesis by remodeling the vascular microenvironment. Paradoxically, NETs can also occlude blood vessels, leading to impaired perfusion and hypoxia. Moreover, NETs upregulate the expression of endothelial adhesion molecules, including ICAM-1, VCAM-1, and E-selectin, which in turn activate endothelial cells and promote neutrophil chemotaxis into the TME. These processes collectively amplify tumor-associated inflammation and support disease progression. (**C**) Immunosuppressive mechanisms. NETs form a physical barrier by coating tumor cells, impeding CD8 + T cell infiltration. NET-associated DNA binds to the transmembrane and coiled-coil domains of CD8^+^ T cells, suppressing their cytotoxic function and inducing apoptosis. In parallel, the release of TGF-β1 by NETs establishes an immunosuppressive feedback loop that further attenuates anti-tumor immunity. Moreover, NETs enhance mitochondrial respiration in CD4⁺ T cells through Toll-like receptor 4 (TLR4) signaling, promoting the differentiation of Tregs and fostering an immune-permissive microenvironment conducive to tumor progression. (**D**) Facilitation of metastasis through molecular signaling. NETs promote tumor metastasis by downregulating E-cadherin and upregulating mesenchymal proteins, thereby promoting epithelial–mesenchymal transition (EMT). High mobility group box 1 (HMGB1) released from NETs binds to the receptor for advanced glycation end products (RAGE) on cancer cells, activating the NF-κB signaling pathway and inducing interleukin-8 (IL-8) secretion. This, in turn, recruits additional neutrophils and sustains NET formation. Following chemotherapy, cancer cells release IL-1β, triggering NET formation. These NETs subsequently activate TGF-β signaling via integrin-αvβ1, enhancing cancer cell chemoresistance and inducing EMT, ultimately promoting cancer cell invasion and dissemination

NETs are increasingly recognized as critical mediators of tumor progression, contributing to immune evasion, angiogenesis, EMT, metastasis, and therapeutic resistance [127, 237]. These chromatin-based structures, comprising DNA, histones, and granule proteins, exert their effects in a spatially restricted and compartment-specific manner, profoundly reshaping the architecture and functional states of the TME. By establishing localized immunosuppressive niches, promoting perivascular remodeling, and reinforcing pre-metastatic microdomains, NETs serve as key orchestrators of the spatiotemporal dynamics that govern tumor evolution and systemic dissemination (Fig. 2).

#### Angiogenesis and vascular modulation

NETs play a pivotal role in modulating tumor vasculature and facilitating metastatic dissemination. First, NETs directly induce endothelial cell apoptosis, resulting in increased vascular permeability and the formation of permissive channels that facilitate tumor cell intravasation and metastatic spread [111]. Second, NET-associated collagenolytic enzymes, including MMP2 and MMP9, degrade basement membrane components, thereby weakening endothelial integrity and reducing cell–cell adhesion. This structural degradation amplifies vascular barrier dysfunction and enhances tumor cell extravasation into distant tissues [15, 111]. Additionally, NETs provoke vascular occlusion and diminished blood perfusion, which activate endothelial cells to upregulate adhesion molecules ICAM-1, VCAM-1, and E-selectin. This pro-inflammatory vascular phenotype promotes leukocyte adhesion and transmigration, further sustaining systemic inflammation and contributing to metastatic niche conditioning [238]. Notably, inflammation during NET formation is accompanied by the release of neutrophil elastase and MMP-9, which cleave laminin and generate bioactive epitopes that activate integrin α3β1 signaling. This reactivation of dormant tumor cells serves as a critical mechanism for metastatic reawakening in established lesions [239].

#### Immune evasion and suppression

NETs substantially contribute to immune evasion by impairing both cytotoxic lymphocyte function and antitumor immune surveillance. First, although CD8⁺ T cells are key effectors of antitumor immunity, NETs have been shown to suppress their infiltration and functional activity. Clinical studies across multiple tumor types have reported a negative correlation between NETs density and CD8⁺ T cell abundance within the TME [240]. In colorectal cancer patients with liver metastases, neutrophil-derived NETs induce exhaustion in both CD4⁺ and CD8⁺ T cells, characterized by upregulation of inhibitory receptors including PD-1, Tim-3, and LAG-3, along with decreased production of effector cytokines such as IL-2, IFN-γ, and TNF-α [127, 241]. These findings underscore the immunosuppressive programming of T cells by tumor-educated NETs. Second, NETs physically encapsulate tumor cells, forming DNA–protein lattices that impede contact-dependent cytotoxicity from CD8⁺ T cells and NK cells [129]. Additionally, NETs DNA interacts with transmembrane and coiled-coil domain 6 on CD8⁺ T cells, suppressing their effector function, promoting apoptosis, and inducing the release of TGF-β1, which further reinforces NET formation and immunosuppression through a positive feedback loop [242].

Furthermore, protease G-mediated cleavage of CCL15 has been implicated in monocyte recruitment, ultimately leading to macrophage accumulation within the TME [237]. This shift in the immune landscape may favor M2 macrophage polarization, reinforcing an immunosuppressive milieu that facilitates tumor progression. NETs also promote Treg differentiation via metabolic reprogramming. In non-alcoholic steatohepatitis models, NETs enhance oxidative phosphorylation in naïve CD4^+^ T cells through TLR4 signaling, driving their differentiation into Tregs and contributing to the establishment of a pro-tumorigenic immune microenvironment [74]. Beyond T cell suppression, NETs exhibit broader immunosuppressive capabilities. The identification of PD-L1 within NETs underscores their capacity to induce T cell exhaustion and impair adaptive antitumor immunity, as evidenced in murine models of hepatic metastasis with ischemia-reperfusion injury [241]. Furthermore, in vitro studies reveal that NETs inhibit NK cell migration and motility, potentially mediated by NET-associated MMP9-mediated suppression of NK cell activity [127, 243].

#### Induction of EMT and metastasis

NETs are increasingly recognized as key facilitators of EMT and metastatic dissemination across multiple tumor types. In triple-negative breast cancer, in vitro studies have shown that NETs significantly enhance tumor cell migration and invasion [244]. Mechanistic insights from breast cancer models reveal that CXCR4⁺CD62L^low^ tumor-associated aged neutrophils initiate mitochondrial permeability transition pore channel opening via the transcription factor SIRT1, leading to mitochondrial DNA release and mitochondria-dependent NET formation in vivo [245]. This process is closely associated with pre-metastatic niche formation, highlighting the translational potential of targeting the SIRT1–aged neutrophil–NETs axis in metastatic breast cancer.

MPO, a key enzymatic driver of NET formation, has been implicated in promoting tumor aggressiveness. Clinically, elevated infiltration of MPO⁺ neutrophils, as assessed by multiplex immunohistochemistry (mIHC), is significantly associated with poor prognosis in patients with colon adenocarcinoma, underscoring the pathological relevance of MPO-dependent NET activity in facilitating metastatic progression and adverse outcomes [67]. In both breast and lung cancer models, NETs incubation with cancer cells downregulates E-cadherin and upregulates mesenchymal proteins, including fibronectin, N-cadherin, and vimentin, collectively driving EMT and facilitating metastatic spread [246, 247].

In hepatocellular carcinoma, Hepatitis B virus-induced S100A9 accelerates NETs generation by activating Toll-like receptor/receptor for advanced glycation end products-ROS signaling [248]. These NETs potentiate hepatocellular carcinoma progression by promoting angiogenesis, facilitating EMT, enhancing MMP-dependent ECM degradation, and entrapping circulating tumor cells, as demonstrated in both in vitro and in vivo models [248].

Furthermore, in glioma, NETs stimulate IL-8 secretion in tumor cells via the NF-κB signaling pathway. IL-8, in turn, recruits additional neutrophils and promotes further NET formation through the PI3K/AKT/ROS cascade, thereby establishing a self-perpetuating pro-metastatic feedback loop [249]. Collectively, these findings position NETs as central orchestrators of metastatic progression, acting through regionally coordinated EMT induction, vascular remodeling, and microenvironmental reprogramming. Their spatiotemporal deployment within the tumor niche enables dynamic modulation of cancer cell behavior and supports the establishment of metastatic competence.

#### Contribution to therapeutic resistance

NETs formation is increasingly recognized as a critical contributor to tumor resistance against conventional therapies. In murine models of breast cancer lung metastasis, chemotherapy, long considered a cornerstone of cancer treatment, paradoxically enhances neutrophil recruitment and NETs production, thereby undermining therapeutic efficacy. Mechanistically, chemotherapy-stressed tumor cells secrete IL-1β, which induces robust NETs formation. These NETs subsequently release integrin αvβ1 and MMP9, activating the TGF-β signaling pathway. TGF-β then drives EMT in tumor cells, reinforcing both chemoresistance and metastatic potential [21].

Given their multifaceted role in tumor progression, NETs have emerged as both a prognostic biomarker and a therapeutic target. Elevated NET levels are positively correlated with poor clinical outcomes across multiple cancer types [127, 250]. Quantitative assessment of NET burden may offer valuable insights into tumor aggressiveness, prognostic stratification, and responsiveness to therapy.

From a therapeutic perspective, targeting NETs-related signaling pathways or inhibiting NETs formation represents a promising strategy to overcome drug resistance and enhance the efficacy of immunotherapy. Given that NETs production is often spatially restricted to perivascular niches, invasive fronts, pre-metastatic niches, hypoxic tumor cores, regions of co-localization with CAFs, and even physical immune-exclusion barrier zones, such interventions may disrupt localized pro-tumor microdomains that support immune evasion [129, 241], EMT [20], and metastatic seeding [111, 113, 245]. By selectively modulating spatially defined NETs–tumor interactions, these strategies could serve as effective adjuncts to standard-of-care regimens and open new avenues for improving clinical outcomes in the context of precision oncology.

## Targeting TANs and NETs in cancer therapy

**Fig. 3:**
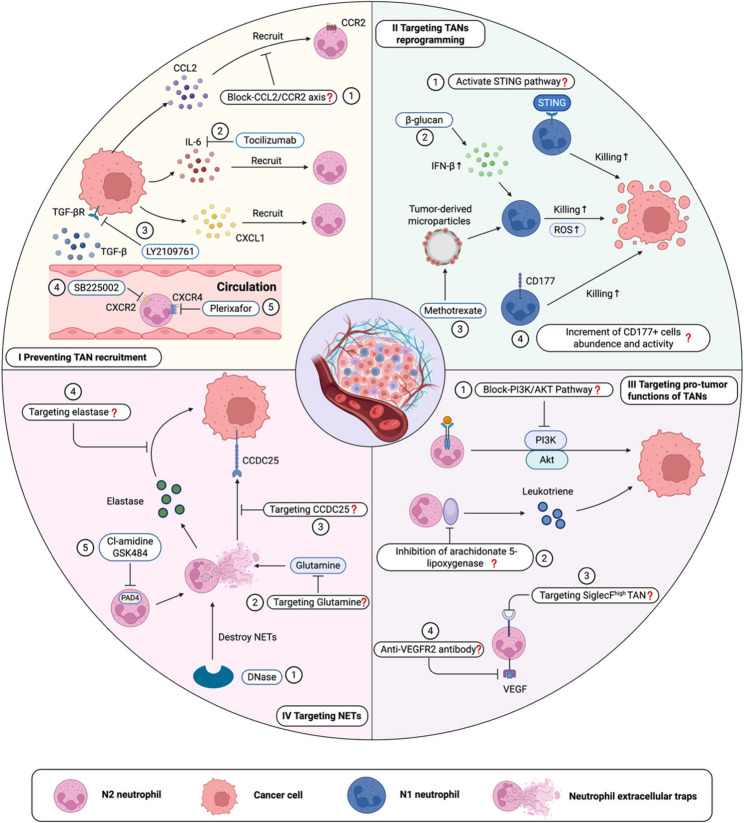
Therapeutic strategies targeting TANs. This figure summarizes current therapeutic strategies aimed at modulating TANs in cancer. These approaches are categorized into four major modalities. Created in https://BioRender.com. (**I**) Preventing TANs Recruitment. This strategy aims to prevent the recruitment of pro-tumorigenic neutrophils (N2-TANs) by targeting key chemokine axes and associated signaling pathways. CXCR2 inhibitors (e.g., SB225002), CXCR4 antagonists (e.g., plerixafor), and IL-6/STAT3 pathway inhibitors (e.g., tocilizumab) have been shown to reduce TAN infiltration while concurrently enhancing CD8⁺ T cell recruitment. Inhibition of TGF-β signaling using receptor antagonists such as LY2109761 disrupts the TGF-β–CXCL1/3–CXCR2 axis, thereby limiting TAN-mediated immunosuppression. Additionally, blockade of the CCL2/CCR2 axis has demonstrated efficacy in suppressing metastatic dissemination. (**II**) Exploiting anti-tumor functions of TANs or reprogramming TANs. This approach promotes the polarization of neutrophils toward an anti-tumorigenic phenotype (N1-TANs) or enhances their intrinsic cytotoxic activity. Methotrexate-induced tumor cell–derived microparticles stimulate neutrophils to exert anti-tumor functions. β-glucan activates reactive oxygen species (ROS)–dependent cytotoxicity through the induction of interferon-β (IFN-β). CD177⁺ neutrophils have been shown to suppress inflammation and enhance anti-tumor immune responses. Furthermore, activation of the stimulator of interferon genes (STING) pathway augments neutrophil-mediated tumor cell killing. (**III**) Targeting pro-tumorigenic TAN subsets or signaling pathways. This strategy aims to eliminate pro-tumorigenic neutrophil subsets or disrupt key signaling pathways that sustain their function. Selective depletion of SiglecF^high^ neutrophils has been shown to inhibit tumor progression. Inhibition of the PI3K/Akt signaling cascade impairs TAN generation and accumulation. Targeting arachidonate 5-lipoxygenase suppresses arachidonic acid synthesis and reduces metastatic potential. Additionally, blockade of vascular endothelial growth factor receptor 2 (VEGFR2) with neutralizing antibodies attenuates the pro-angiogenic activity of neutrophils. (**IV**) Targeting NETs.Targeting the formation or degradation of NETs represents a promising strategy to inhibit tumor progression. Inhibition of CCDC25 signaling or metabolic pathways such as glutamine and fatty acid synthesis attenuates NETs-mediated metastasis. Peptidylarginine deiminase 4 (PAD4) inhibitors (e.g., Cl-amidine) suppress NETs formation, while recombinant DNase I enzymatically degrades extracellular DNA scaffolds. Neutrophil elastase, a key mediator of NET-associated metastatic activity, has emerged as a compelling therapeutic target, with neutrophil elastase inhibitors showing potential for limiting NET-driven tumor dissemination

The spatiotemporal heterogeneity of TANs and the paradoxical functions of NETs in promoting metastasis and immune suppression pose substantial challenges to the development of targeted therapeutic strategies. Nevertheless, these complexities also reveal critical opportunities for precision immunomodulation. In this section, we highlight several promising intervention approaches (Fig. 3; Table 3). These include inhibiting TAN recruitment by disrupting chemokine signaling pathways; reprogramming pro-tumorigenic phenotypes via targeted modulation of intracellular signaling cascades; selectively targeting distinct TAN subpopulations using single-cell and spatial omics platforms; and suppressing NETs formation, promoting NETs clearance, or neutralizing their bioactive components. Collectively, these emerging strategies aim to constrain TAN/NETs-mediated tumor support and enhance the efficacy of immunotherapy.

**Table 3:** Potential therapeutic strategies targeting TANs

Targeting	Treatment strategy/mechanism	Drug name	Cancer type	Clinical trial number	References
TANs recruitment	Block CXCLs/CXCR2 axis	SB225002	lung cancer		[42]
TANs recruitment	Inhibit CXCR1/2	Navarixin	NSCLC(IV)	NCT03473925	
TANs recruitment	Inhibit CXCR1/2	SX-682	NSCLC (IIIC/IV)	NCT05570825	-
TANs recruitment	Block CXCR2/CXCL1 signaling pathway	CXCR2 blocking Ab	Pancreatic cancer	-	[39]
TANs recruitment	Inhibit CXCR4	Plerixafor	Ovarian cancer	-	[43]
TANs recruitment	Block Gas6/AXL axis	Bemcentinib	PDAC liver metastasis	NCT03649321	[251]
TANs recruitment	Block CCL2/CCR2 axis	-	Breast cancer	-	[252]
TANs recruitment	Inhibit CXCR2	AZD5069	CRC	-	[162]
TANs recruitment	Block TGFβ/SMAD3 signaling	LY2109761	CRC liver metastasis	-	[125]
TANs recruitment	Inhibit cathepsin C secretion	AZD7986	Breast cancer	NCT03218917^*^	[122]
TANs recruitment	Block IL-1β signaling	IL-1β neutralizing Ab	Breast cancer	-	[122]
TANs recruitment	Block pleiotrophin signaling	3B10 (anti-pleiotrophin mAb)	Breast cancer	-	[165]
TANs reprogramming	Activate STING pathway	5,6-dimethylxanthenone-4-acetic acid (STING agonist)	Breast cancer	-	[252]
NETs inhibition	Inhibit PAD4	Cl-amidine, GSK484	Multiple cancers	-	[60, 253]
NETs inhibition	Block NET-DNA interaction	DNase I	CRC	-	[254]
Immune checkpoint	Block CD47-SIRPα axis	SIRPα mAb	Burkitt’s lymphoma	-	[255]
Function of neutrophils	Block IL6-STAT3-PD-L1 immunosuppressive pathway	-	HCC	-	[202]
Function of neutrophils	Inhibit fatty acid transport protein 2	Lipofermata	Multiple cancers	-	[148]
Combination therapy	Combination of anti-CXCL5 and anti-PD-L1 treatment	Anti-CXCL5 Ab	Lung cancer	-	[150]

Abbreviation: *TANs* tumor-associated neutrophils,* NSCLC* non-small cell lung cancer, *PDAC* pancreatic ductal adenocarcinoma, *CRC* colorectal cancer, *NETs* neutrophil extracellular traps, *PAD4* peptidyl arginine deiminase 4, *HCC* hepatocellular carcinoma

^*^The clinical trial was conducted in inflammatory lung disease

### Innovative strategies for TAN modulation

Currently, therapeutic strategies targeting TANs remain limited compared to interventions aimed at other immune or stromal components within the TME. This discrepancy arises in part from the rapid turnover and compensatory recovery mechanisms intrinsic to neutrophils, which challenge the durability of therapeutic effects [256]. Moreover, the pronounced spatiotemporal heterogeneity of TANs adds substantial complexity. TANs comprise distinct functional subsets that vary by anatomical location and tumor developmental stage, ranging from anti-tumorigenic phenotypes to pro-tumorigenic populations that facilitate tumor progression and metastasis [24, 25, 30, 44]. This heterogeneity significantly complicates the design of effective, broadly applicable targeting strategies. However, advancing technologies such as single-cell transcriptomics and spatial omics now allow for more refined characterization of TAN subsets. A comprehensive understanding of their spatial distribution, developmental trajectories, and phenotypic markers is critical for the rational design of targeted therapies. Through precise molecular profiling, it becomes possible to identify TAN populations that exert context-dependent effects, enabling the development of spatiotemporally informed interventions that either deplete pro-tumor subsets or promote the persistence of protective phenotypes. To this end, we summarize below the principal strategies currently under investigation for modulating TAN recruitment, polarization, and effector function in the context of cancer therapy.

#### Targeting TANs recruitment

As key inflammatory mediators, TANs are actively recruited into the TME by various chemokines and inflammatory signals, where they often promote tumor progression. Inhibiting TAN infiltration into the TME has therefore emerged as a promising therapeutic strategy, primarily through the blockade of chemokine pathways involved in their recruitment.

One of the most well-studied axes is the CXCL/CXCR2 signaling pathway. TANs, which are highly enriched in lung cancer tissues and associated with poor prognosis, promote tumor proliferation, apoptosis resistance, senescence, and EMT via a CXCLs/CXCR2 autocrine loop. Pharmacologic inhibition of CXCR2 using SB225002 suppresses tumor growth by impairing TAN recruitment, inducing tumor cell apoptosis and senescence, enhancing CD8⁺ T cell activation, and improving the efficacy of cisplatin chemotherapy [42]. Similar effects have been observed in pancreatic, and other tumor models [26, 125, 257, 258]. Relevant clinical trials are in progress, such as NCT04477343 [26, 259] and NCT03161431 [258, 260]. In a model of in situ pancreatic ductal adenocarcinoma, inhibition of CXCR2 prevented neutrophil mobilization from the circulation, thereby enhancing the efficacy of folfirinox [261]. In patients with advanced solid cancers, the CXCR1/2 antagonist navarixin has been evaluated in combination with pembrolizumab for stage IV disease (NCT03473925) [262, 263]. Although this regimen demonstrated favorable safety and tolerability, its clinical efficacy was limited [262]. Importantly, systemic CXCR2 blockade may compromise innate host defense, underscoring the need for tumor-selective or spatially restricted therapeutic approaches.

Beyond CXCR2, several alternative chemokine axes have been explored. In a murine ovarian cancer model, the CXCR4 antagonist plerixafor in combination with a PD-1 inhibitor promoted depletion of MDSCs and enhanced CD8⁺ T cell infiltration [43]. Ulocuplumab, an anti‑CXCR4 monoclonal antibody, showed an acceptable safety profile and high response rates in Ib/II trials of relapsed or refractory multiple myeloma [264]. Moreover, inhibition of tumor glutamine metabolism has been shown to suppress G-CSF production, limit MDSC mobilization, and promote tumor cell apoptosis [265]. Targeting the CCL2/CCR2 axis has emerged as a promising therapeutic strategy. Tumor-derived CCL2 facilitates the recruitment of CCR2⁺ immune effector monocytes to the lungs, where they augment the antitumor activity of neutrophils, collaboratively suppressing metastatic progression. Modulation of this axis may potentiate these immune responses and prevent tumor cell colonization and outgrowth at distant metastatic sites [252].

Targeting TANs may also offer therapeutic leverage in overcoming drug resistance and preventing tumor recurrence. In a murine model of pancreatic ductal adenocarcinoma with liver metastasis, gemcitabine treatment induced only transient tumor control. Upon cessation, metastatic tumor cells upregulated the secretion of CXCL1 and CXCL2, thereby driving CXCR2-dependent neutrophil recruitment. These recruited neutrophils expressed growth arrest–specific 6, which in turn activated AXL receptors on tumor cells, promoting metastatic outgrowth [251]. Notably, this neutrophil–tumor interaction was spatially confined to metastatic niches, highlighting the significance of local microenvironmental cues in shaping therapeutic escape. Combined inhibition of the CXCR2 and GAS6/AXL axes alongside chemotherapy has been shown to disrupt this pro-metastatic loop and suppress relapse [251].

#### Reprogramming TAN phenotypes

Given the pronounced spatiotemporal heterogeneity of TANs, selectively enhancing antitumor subpopulations offers a promising therapeutic avenue. Certain TAN subsets, such as anti-tumorigenic TANs, exhibit cytotoxicity and immune-stimulatory functions. Strategies aimed at promoting their expansion and function could transform the immunosuppressive TME and improve therapeutic efficacy.

Recent studies have demonstrated that tumor cell–derived particles induced by methotrexate act as immunostimulatory agents that reprogram neutrophils toward an anti-tumorigenic phenotype. These reprogrammed TANs enhance T cell–mediated antitumor responses and significantly increase the efficacy of anti–PD-L1 therapy [266]. Similarly, exposure to IFN-β drives the polarization of TANs into tumoricidal states [252]. In addition, β-glucan has been shown to promote anti-tumorigenic TANs through an involving type I interferon signaling [267]. Notably, this trained immunity phenotype is transferable: antitumor neutrophil properties can be conferred to naïve recipients through bone marrow transplantation, indicating its great potential for clinical translation [267].

Furthermore, the antitumor activity of neutrophils can be directly potentiated using cytokines or small-molecule agents. For example, IFN-β has been shown to enhance neutrophil-mediated cytotoxicity against tumor cells [41]. Conversely, the tumor-promoting functions of neutrophils can be suppressed by pharmacologic agents that interfere with the release or activity of immunosuppressive mediators. A representative agent is the COX-2 inhibitor celecoxib, which has been explored for its capacity to attenuate neutrophil-derived pro-tumorigenic signals and thereby enhance the efficacy of immunotherapy [268]. These findings underscore the feasibility of manipulating TAN phenotypes with pharmacological precision.

With the growing application of single-cell sequencing technology and spatial genomics, increasing granularity in the classification of TAN subtypes is being achieved. The identification and functional characterization of tumor-suppressive neutrophil subpopulations represents an emerging therapeutic opportunity. Notably, CD177⁺ neutrophils have been shown to exert protective roles by limiting epithelial proliferation, dampening inflammatory signaling, and fostering antitumor immunity. Enhancing the abundance or function of this subset may offer a viable strategy for tumor control [81].

Targeting neutrophil-specific phagocytosis checkpoints also holds promise. Signal regulatory protein α SIRPα, highly expressed on neutrophils, binds to CD47 on tumor cells, delivering a “do not eat me” signal that restricts neutrophil-mediated cytotoxicity. In transgenic mouse models expressing human SIRPα, co-administration of a SIRPα-targeting monoclonal antibody with an anti-tumor antibody has been shown to enhance neutrophil-mediated elimination of tumor cells [255]. Blockade of the CD47–SIRPα axis disrupts this inhibitory phagocytic checkpoint and augments neutrophil cytotoxicity against antibody-coated tumor targets [8, 269]. Early clinical trials of CD47–SIRPα inhibitors (Hu5F9-G4, IBI188; NCT02216409, NCT03717103) have demonstrated acceptable safety profiles, and ongoing studies are evaluating bispecific antibodies that simultaneously target CD47–SIRPα and PD-1/PD-L1 pathways in solid tumors (NCT05200013, NCT05731752, NCT05780307, NCT05048160) [270, 271].

In parallel, TMEM173 (also known as STING) has emerged as a key intracellular regulator of neutrophil function. Elevated STING expression in TANs is associated with enhanced antitumor activity, and pharmacologic activation of the STING pathway has been shown to boost neutrophil cytotoxicity while suppressing metastatic dissemination [252]. Additionally, modulation of the angiotensin-converting enzyme and angiotensin II type 1 receptor, nicotinamide phosphoribosyltransferase, or the chemokine receptor CXCR4 has been shown to shift TANs toward a tumor-inhibitory state in murine models [272–274]. These approaches, grounded in growing insights into TAN heterogeneity, highlight the therapeutic potential of selectively reprogramming neutrophil subsets.

Microbial-based strategies offer a unique modality to re-educate TANs. Treatment with Staphylococcus aureus bioparticles or Mycobacterium bovis Bacillus Calmette Guerin has been reported to shift the TME from a chronic inflammatory state to an acute microbial inflammatory milieu. This immune reconditioning facilitates the recruitment and activation of neutrophils, driving their phenotypic conversion from a pro-tumorigenic to an anti-tumorigenic state, thereby suppressing tumor growth [275]. However, it is important to note that this approach is not without its limitations. The efficacy of microbial therapy may be contingent on repeated stimulation, which may provoke excessive inflammatory responses or immune-related adverse events. These limitations underscore the need for precise modulation strategies that preserve therapeutic efficacy while minimizing systemic toxicity.

Metabolic reprogramming also represents a critical determinant of TAN function. Targeting glycolytic and lipid metabolic pathways can enhance neutrophil antitumor activity. Inhibition of lactate production or disruption of HIF-1α signaling suppresses pancreatic ductal adenocarcinoma progression [276], whereas selective blockade of long-chain fatty-acid transport, using the FATP2 inhibitor lipofermata, abrogates TAN-mediated immunosuppression and synergizes with anti-PD-1 therapy to improve survival [148, 277]. Likewise, systemic administration of the glutaminase inhibitor JHU083 in 4T1 breast cancer reduces G-CSF production, diminishes MDSC recruitment, and induces apoptosis within intratumoral and circulating myeloid compartments [265].

Collectively, these studies underscore the therapeutic promise of modulating TAN phenotypes as a means of redirecting neutrophil plasticity toward antitumor immunity. By integrating spatial transcriptomics, single-cell technologies, and immune landscape profiling, it becomes increasingly feasible to identify functionally distinct TAN subsets, track their dynamic evolution across tumor regions and disease stages, and design targeted strategies that either potentiate tumor-suppressive TANs or neutralize pro-tumor counterparts. This phenotypic reprogramming, whether induced by immunostimulatory agents, metabolic modulators, or microbial mimetics, may complement immune checkpoint blockade and other frontline therapies. Ultimately, spatially informed manipulation of TAN subpopulations represents a next-generation approach to reshaping the immunosuppressive TME and overcoming resistance in solid tumors.

#### Targeting specific TAN subpopulations

As the functional heterogeneity of TANs becomes increasingly apparent, precision targeting of tumor-promoting TAN subsets emerges as a promising therapeutic direction. These subpopulations, shaped by tumor-derived factors and local microenvironmental cues, contribute to immune suppression, invasion, and metastasis via distinct molecular programs.

One approach involves directly targeting effector molecules uniquely expressed by pro-tumorigenic TANs. AGR2, secreted by TANs in colorectal cancer, has been identified as a key driver of metastatic dissemination. Functional blockade of AGR2 using neutralizing antibodies or gene silencing techniques significantly impairs tumor cell migration and invasion [158, 278]. Similarly, SiglecF^high^ neutrophils have been implicated in promoting tumor progression. Their selective depletion or functional inhibition via antibody-based strategies represents a compelling avenue for therapeutic intervention [142]. In parallel, the soluble receptor for advanced glycation end-products (sRAGE), associated with tumor-induced bone remodeling and systemic neutrophil responses, has been proposed as a targetable mediator of neutrophil-driven tumorigenesis. Inhibitors or neutralizing antibodies against sRAGE may mitigate these remote effects and limit cancer-associated inflammation [142].

Beyond direct effector targeting, inhibiting upstream signaling cascades that drive pro-tumorigenic polarization may curtail the emergence of tumor-supportive TAN phenotypes. For instance, by impeding the PI3K/Akt and p38/MAPK signaling pathways, it is possible to prevent normal neutrophils from acquiring TAN-like properties in response to stimulation by hepatocellular carcinoma cells, thereby reducing the formation of TANs [102]. Likewise, KIAA1199, a tumor-secreted factor that promotes TAN recruitment, can be silenced genetically or pharmacologically via pirfenidone to reduce neutrophil infiltration, enhance CD8⁺ T cell–mediated immunity, and suppress liver metastasis [125]. Additional studies have implicated neutrophil-derived leukotrienes in facilitating metastatic colonization. Pharmacologic inhibition of leukotriene biosynthesis, such as via arachidonate 5-lipoxygenase inhibitors, has demonstrated efficacy in restraining metastatic progression in murine breast cancer models [279]. Moreover, breast tumors can reprogram hematopoietic stem cells through IL-1β–driven myelopoiesis, fostering the emergence of immunosuppressive neutrophil progenitors early in development. Therapeutic blockade of IL-1β reverses this myeloid skewing, normalizes neutrophil differentiation, and reduces metastatic burden, highlighting IL-1β as a clinically relevant intervention target [280].

Despite these advances, many of the proposed targets remain in preclinical or conceptual stages. The translational development of effective inhibitors will require substantial investment and mechanistic validation. A critical objective for future studies will be to delineate and preserve beneficial TAN subtypes while selectively suppressing deleterious subsets [281]. This nuanced approach will likely benefit from emerging technologies in spatial transcriptomics and single-cell profiling, enabling precise characterization of TAN sublineages within diverse tumor contexts. As these tools mature, they may unlock new opportunities for neutrophil-centered immunomodulation in cancer therapy.

#### Modulating cytokines that shape TAN responses

Cytokines play a central role in orchestrating the recruitment, polarization, and effector functions of TANs within the TME. Targeting specific cytokine-mediated signaling axes thus represents a viable strategy to suppress protumorigenic TAN activity and reshape the immunologic landscape toward a more favorable therapeutic milieu.

##### IL-6

IL-6 is a key driver of neutrophils recruitment and polarization [8, 282]. IL-6 not only promotes TAN expansion but also fosters immune suppression by inhibiting NK cell activity via activation of the STAT3 signaling pathway and skewing neutrophil precursors toward immunosuppressive phenotypes. Pharmacologic blockade of this pathway using the IL-6 receptor antibody tocilizumab has demonstrated reversal of these effects and enhancement of T cell–mediated antitumor immunity in preclinical models [103]. Moreover, dual blockade of IL-6 and PD-L1 significantly reduced tumor burden and prolonged survival in a heterotopic hepatocellular carcinoma model [283]. Icariin, a small molecule of traditional Chinese medicine origin has also been shown to inhibit the IL-6/JAK2/STAT3 axis. In Phase I and II clinical trials involving hepatocellular carcinoma patients, icariin exhibited immunomodulatory activity, including suppression of neutrophil-mediated tumor promotion and enhancement of adaptive immune responses [284–286]. These findings support IL-6/STAT3 as a promising dual immunoregulatory and neutrophil-targeting axis.

##### TGF-β

Elevated TGF-β levels in the TME correlate with increased neutrophil infiltration and poorer clinical outcomes across cancer types [2, 147, 158]. Inhibition of the TGF-β receptor with LY2109761 has been shown to block KIAA1199-mediated neutrophil recruitment and suppress immunosuppressive activity, thereby limiting liver metastasis in colorectal cancer models [125]. ALK5 inhibitors (e.g., AZ12601011) and anti-TGF-β monoclonal antibodies (e.g., 1D11) have further demonstrated efficacy in reducing TAN infiltration and reversing immunosuppressive networks through inhibition of TGF-β signaling. In the KPN mouse model, these agents significantly decreased TAN density in hepatic metastases while enhancing T cell infiltration and effector function [39, 162].

Clinically, the anti-TGF-β antibody NIS793 inhibits tumor-cell TGF-β signaling and remodels the TME to restore immune surveillance; it is under active evaluation in colorectal and non–small cell lung cancer trials [287]. Furthermore, the multi-kinase inhibitor lenvatinib, which suppresses TGF-β signaling, synergizes with PD-1 blockade in HCC by reprogramming the immune landscape [288]. However, emerging data have revealed mechanistic complexity. Contradictory findings indicate that lenvatinib monotherapy promotes neutrophil infiltration via induction of CXCL2 and CXCL5, and upregulates PD-L1 expression in neutrophils through tumor-derived lactate-mediated activation of the MCT1/NF-κB/COX-2 pathway, ultimately fostering an immunosuppressive TME [185]. This apparent dichotomy underscores the context-dependent immunomodulatory effects of lenvatinib. Notably, targeted inhibition of COX-2 to suppress PD-L1 + neutrophils has been proposed as a promising strategy to complement lenvatinib-based therapies [185]. Consequently, the selection of combination therapeutic targets should comprehensively address TME heterogeneity, extending beyond dual-agent regimens to systematically evaluate the potential of multi-pathway targeting strategies for enhanced therapeutic synergy.

##### GM-CSF

GM-CSF is another pivotal cytokine involved in TAN education. It promotes neutrophil-derived oncostatin M secretion, which in turn activates the JAK/STAT pathway in tumor cells to drive VEGF production and angiogenesis [195]. Additionally, GM-CSF, released by TANs, has been shown to induces PD-L1 expression on tumor cells in the lung cancer microenvironment, thereby suppressing CD8⁺ T cell cytotoxicity [150]. Therefore, neutralization of GM-CSF using monoclonal antibodies may counteract these immunosuppressive effects and restore effective antitumor T cell responses.

Taken together, these cytokine-targeted approaches hold substantial promise for interrupting the paracrine loops that sustain TAN-mediated tumor progression. Rational combination strategies, such as integrating cytokine blockade with immune checkpoint inhibition or chemotherapeutic regimens, are likely to enhance overall treatment efficacy while mitigating TAN-driven resistance mechanisms.

#### Integrating microenvironmental factors

The spatial distribution, phenotypic polarization, and functional heterogeneity of TANs are profoundly shaped by their interactions with key components of the TME. Among these, VEGF signaling and hypoxia represent pivotal microenvironmental cues that orchestrate TAN dynamics and contribute to tumor progression. Targeting these pathways, particularly in combination with conventional therapies, offers promising avenues to overcome neutrophil-mediated resistance and enhance antitumor efficacy.

##### VEGF Axis

VEGF functions as a central mediator of tumor angiogenesis and exerts bidirectional influence on TAN behavior. On one hand, VEGF induces the recruitment of proangiogenic neutrophils to the regions with high VEGF expression [45]. On the other hand, TANs themselves secrete VEGF upon activation, further amplifying angiogenic signaling and promoting metastatic dissemination [119]. Therapeutic blockade of this feedback loop has shown promise. For example, sorafenib, a multi-kinase inhibitor with anti-angiogenic properties, has been widely utilized in the treatment of advanced malignancies such as hepatocellular carcinoma. Its mechanism involves inhibition of VEGF signaling, leading to suppression of tumor-induced neovascularization. However, emerging evidence suggests that sorafenib treatment is paradoxically associated with increased accumulation of TANs, a phenomenon linked to poor clinical outcomes [102, 289]. Notably, recent studies have shown that deficiency in IL-1β signaling mitigates TAN accumulation following anti-angiogenic therapy [123], highlighting the potential benefit of rational combination strategies. Integrating IL-1β blockade with VEGF-targeted therapy may more effectively suppress TAN-mediated resistance and enhance anti-angiogenic efficacy.

In addition, preclinical studies have demonstrated that neutrophil depletion can synergize with sorafenib to enhance therapeutic efficacy. In murine tumor models, co-administration of sorafenib with an anti-Ly6G antibody significantly inhibited tumor growth and angiogenesis, suggesting that TANs may counteract the anti-tumor effects of anti-angiogenic therapy by sustaining a pro-angiogenic and immunosuppressive niche [102]. Notably, in a pancreatic ductal adenocarcinoma model, a distinct subset of neutrophils, T3-TANs characterized by pro-angiogenic gene expression, was shown to accelerate tumor progression. Anti-VEGF therapy effectively suppressed the angiogenic activity of these T3-TANs, highlighting the relevance of spatially and functionally distinct neutrophil subsets in shaping therapeutic outcomes [30].

##### Hypoxia

Hypoxia represents a hallmark of the TME and serves as a potent driver of TAN accumulation and polarization [55]. Hypoxic regions within tumors frequently overlap with immune exclusion zones and angiogenic niches, wherein TANs adopt pro-tumorigenic phenotypes that support immune evasion, neovascularization, and therapeutic resistance. Thus, reversing tumor hypoxia has emerged as a promising strategy to modulate neutrophil behavior and reprogram the immunosuppressive TME. In a preclinical hepatocellular carcinoma model, pharmacologic targeting of hypoxia using the novel HIF inhibitor 32-134D effectively disrupted hypoxia-adaptive signaling. This intervention led to reduced recruitment of myeloid-derived suppressor cells from the bone marrow, downregulation of pro-angiogenic gene expression, and attenuation of key immunosuppressive cellular components within the TME. Notably, when combined with PD-1 blockade, HIF inhibition significantly improved tumor clearance, increasing the tumor eradication rate from 25% to 67% [290]. These findings underscore the therapeutic potential of integrating hypoxia-targeted agents with immunotherapy to remodel TAN-infiltrated niches and overcome resistance mechanisms. Future strategies should consider the spatial topography of TAN accumulation in hypoxic cores and perivascular regions to guide rational combinatorial designs.

Collectively, these insights highlight the critical influence of microenvironmental cues, particularly VEGF-driven angiogenesis and tumor hypoxia, on the spatial patterning, phenotypic polarization, and functional reprogramming of TANs. These factors not only facilitate immune exclusion and metastatic seeding but also compromise the efficacy of conventional and immune-based therapies. Therapeutic strategies that target VEGF signaling and HIF-mediated pathways, especially when rationally combined with immune checkpoint blockade or neutrophil-modulating agents, offer a compelling opportunity to dismantle TAN-enriched immunosuppressive niches. As technologies such as spatial transcriptomics and intravital imaging continue to refine our understanding of TAN localization and microdomain-specific behavior, the development of spatially guided combination therapies will be pivotal in overcoming neutrophil-driven therapeutic resistance and achieving durable antitumor responses.

#### Engineering strategies

Leveraging their intrinsic chemotactic and inflammatory-homing properties, neutrophils have emerged as innovative vehicles for targeted drug delivery within inflamed and tumor tissues, including the central nervous system. Their natural ability to navigate through complex and hypoxic TME positions them as ideal candidates for precision delivery of therapeutic agents.

A representative strategy involves loading chemotherapeutics, such as paclitaxel or doxorubicin—into liposomes or nanoparticles, which are subsequently internalized by neutrophils. Upon reaching inflamed or postoperative tumor sites, these engineered neutrophils release their drug payload in response to inflammatory cues, thereby effectively suppressing glioma recurrence in murine models [291]. This inflammation-responsive delivery mechanism minimizes systemic toxicity while ensuring spatiotemporally controlled drug release.

Beyond synthetic nanocarriers, bacterial particles have been harnessed as dual-function platforms that act both as drug carriers and vascular disruptors. Upon administration, these particles elicit local immune activation, triggering neutrophil recruitment and phagocytosis. The engulfed particles transform neutrophils into biological microrobots, capable of penetrating deep into otherwise inaccessible tumor cores and delivering therapeutic agents directly within hypoxic or necrotic zones, thereby amplifying antitumor efficacy [292].

Further advances in nanobiotechnology have equipped neutrophils with photocatalytic nanoparticles such as Fe₃O₄@TiO₂, which generate ROS upon photoactivation. This enhances both antimicrobial defense and antitumor immune responses, illustrating the potential for multifunctional therapeutic systems [293]. Moreover, neutrophils have been engineered to carry urease-powered micromotors, enabling active propulsion for targeted thrombolysis, further underscoring their versatility as dynamic drug delivery platforms [294].

Collectively, these engineering approaches demonstrate the feasibility of exploiting neutrophils as living delivery vehicles that integrate biological sensing with controlled release functions. As advances in biomaterials and cellular engineering continue to converge, neutrophil-based delivery systems may evolve into next-generation therapeutic platforms capable of precise, responsive, and deep-tissue drug delivery in oncology and beyond.

### Exploiting NETs for immunotherapy

Given the multifaceted role of NETs in promoting tumor progression, including immune evasion, EMT, metastatic niche formation, and therapeutic resistance, targeting NETs represents a compelling immunotherapeutic strategy. Although current approaches remain limited, ongoing research offers promising avenues for translational advancement.

#### Inhibiting NET formation

NETs are increasingly recognized as critical facilitators of tumor progression through their ability to promote EMT, remodel the ECM, and establish pro-metastatic niches [20, 237]. In pancreatic ductal adenocarcinoma, for example, NETs have been implicated in the early steps of liver metastasis, and early intervention with NETs inhibitors has been shown to effectively prevent metastatic outgrowth [295]. The efficacy of this strategy has been demonstrated by current relevant studies. In breast cancer models, tumor cells adhere to NETs via the transmembrane receptor CCDC25, and blockade of CCDC25 significantly reduces both primary tumor burden and distant metastasis [16].

Emerging studies further reveal that TANs undergo metabolic reprogramming toward glutamine and fatty acid dependence to sustain NETs production. Pharmacologic inhibition of these metabolic pathways markedly suppresses NETs formation [38, 188]. At the molecular level, the enzyme PAD4 mediates histone citrullination and plays a key role in the formation of NETs. PAD4 inhibitors such as Cl-amidine and GSK484 have demonstrated efficacy in blocking NETs release, while naturally occurring agents like epigallocatechin-3-gallate and DHT have also been shown to impede NETs formation and inhibit tumor cell migration and invasion [60, 237, 253].

Cytokine-mediated pathways also play a central role. Tumor-derived IL-8 has been identified as a potent inducer of NETs formation via CXCR2 signaling. Notably, dual inhibition of CXCR1 and CXCR2 with the small-molecule antagonist Reparixin significantly attenuated intratumoral NETs deposition in breast cancer-bearing mice [129]. Additionally, in the prostate cancer, PSMA1 activates the NF-κB signaling pathway to induce HIF-1α expression, forming a PSMA1–NF-κB–HIF-1α axis that drives TANs toward pro-tumor functional polarization and promotes the synthesis and release of NETs. Targeted inhibition of PSMA1 effectively blocks this signaling axis, reduces NET formation, and reverses the immunosuppressive microenvironment, thereby suppressing tumor progression [296].

Collectively, these findings underscore the therapeutic potential of targeting NETs formation at both early and advanced stages of tumor progression. Inhibiting NETs release through metabolic blockade, enzymatic inhibition, or cytokine receptor antagonism may serve as a strategy to disrupt neutrophil-driven metastatic dissemination and improve patient survival, particularly in spatially defined pro-metastatic niches such as invasive fronts, pre-metastatic sites, and hypoxic tumor cores.

#### Clearing pre-existing NETs

Given the critical role of NETs in tumor progression, direct degradation of pre-formed NETs represents a rational therapeutic approach to neutralize their protumorigenic effects. One of the most well-studied strategies involves the use of DNase I, which enzymatically cleaves the DNA backbone of NETs. In preclinical models of spontaneous pancreatic ductal adenocarcinoma and experimental liver metastases, daily intraperitoneal administration of DNase I significantly reduced the development of liver metastatic lesions. Notably, DNase I treatment also decreased the abundance of activated CAFs within micrometastatic foci, thereby inhibiting NETs-mediated metastasis [295]. To improve delivery specificity and therapeutic efficacy, a hybrid liposomal delivery system has been engineered to encapsulate DNase I and incorporate a CCDC25 DNA sensor within its membrane. This design facilitates targeted localization to NETs structures, enabling spatially restricted degradation of NETs [254].

However, DNase I primarily targets the extracellular DNA scaffold of NETs but can only partially inhibit the activity of neutrophil elastase and reduce some of the damage [297]. This limitation underscores the need for combinatorial approaches that simultaneously dismantle the structural components of NETs and neutralize their bioactive mediators.

#### Eliminate NETs active ingredients

NETs mediate their tumor-promoting effects through a repertoire of proteolytic enzymes, including neutrophil elastase, proteinase G, and MMPs, which are capable of degrading ECM, remodeling tissue architecture, and modulating immune responses [15, 239, 298]. Pharmacological strategies aimed at neutralizing these enzymatic constituents have demonstrated potential in attenuating tumor progression and improving clinical outcomes. Among these, neutrophil elastase has received particular attention, as its overexpression correlates with poor prognosis in breast cancer. Inhibition of neutrophil elastase activity may help reduce tumor metastasis [39].

Sivelestat, a small-molecule inhibitor of neutrophil elastase approved in Japan for the treatment of acute respiratory distress syndrome, has attracted interest as a potential reference agent for oncological applications. However, its limited efficacy in cancer settings may be attributable to the existence of neutrophil elastase -independent mechanisms of NET formation [237, 299, 300]. This highlights the mechanistic complexity of NETs biology and underscores the necessity of broader-spectrum or combinatorial therapeutic strategies that concurrently target multiple NETs-related effector pathways.

Despite encouraging preclinical data, clinically validated NETs-targeted therapies remain scarce. Ongoing investigations are evaluating the integration of NETs inhibitors with standard-of-care cancer therapies, such as chemotherapy, immune checkpoint blockade, or anti-angiogenic agents. Nevertheless, therapeutic targeting of NETs presents unique challenges. NETs serve a dual role in host biology, while they facilitate tumor progression and immune evasion, they are also integral to innate immune defense. Indiscriminate inhibition of NETs may therefore compromise antimicrobial surveillance or inadvertently dampen antitumor immunity under specific conditions [301].

Moreover, the functional roles of NETs appear to be context-dependent, varying across tumor types and individual patients. This heterogeneity suggests that therapeutic interventions targeting NETs may yield clinical benefit only in specific patient subgroups, thereby necessitating further investigation into the underlying mechanisms and differential therapeutic efficacy across distinct cancer types [73]. Emerging evidence also points to the existence of NETs subtypes with distinct molecular and functional profiles, further complicating therapeutic design. Therefore, achieving precise and effective NETs-directed interventions will require comprehensive molecular characterization of NETs structures, their cellular origins, and the upstream and downstream regulatory circuits that govern their formation and activity.

### Challenges and future directions

Despite encouraging advances in our understanding of TANs and NETs, the clinical translation of targeting strategies remains in its infancy. Several major challenges must be addressed to fully harness the therapeutic potential of TAN- and NETs-targeted interventions.

First, the inherent spatiotemporal heterogeneity of TANs complicates classification, biomarker development, and therapeutic targeting. TAN phenotypes exhibit considerable plasticity, varying across tumor types, disease stages, and spatial niches within the TME. While the N1–N2 paradigm provides a foundational framework, emerging evidence highlights a spectrum of intermediate or hybrid states with overlapping features. Addressing this complexity requires high-dimensional profiling strategies, such as spatial transcriptomics and single-cell multi-omics, to achieve more granular subtype delineation and functional annotation.

Second, the mechanistic basis of TAN plasticity remains incompletely defined. Tumor-derived cytokines, metabolic reprogramming, and hypoxia are known contributors, but their integrated influence within distinct microenvironmental contexts is poorly understood. Elucidating these context-specific regulatory networks is essential for guiding targeted reprogramming strategies that convert TANs toward tumor-suppressive states without impairing innate host defenses. Moreover, therapeutic modulation of TANs must consider potential synergistic or antagonistic effects with concurrent immunotherapies.

Third, NETs-targeted therapies face the dual challenge of functional heterogeneity and physiological necessity. While NETs contribute to tumor progression through the promotion of immune evasion, angiogenesis, and metastasis, they also serve essential roles in host defense and tissue repair. Global suppression of NETs formation may inadvertently increase susceptibility to infections or compromise tissue homeostasis. Furthermore, NETs are not uniform entities: they differ in cellular origin, molecular composition, and immunological function. A more refined molecular characterization of NETs subtypes, distinguishing tumor-promoting from potentially protective forms, will be crucial for precision targeting.

Another significant barrier is the lack of robust, clinically validated biomarkers for assessing TAN or NETs activity. Existing approaches are largely limited to tissue-based observations, which are invasive and often retrospective. There is a pressing need for dynamic, non-invasive biomarkers, such as NETs-associated circulating DNA, neutrophil-derived extracellular vesicles, and spatially defined transcriptomic signatures, to facilitate real-time patient stratification, therapy monitoring, and treatment optimization.

Looking forward, future research must prioritize the integration of TAN- and NETs-directed therapies into multidimensional treatment strategies. Rational combination regimens, incorporating ICIs, anti-angiogenic agents, metabolic reprogramming tools, and microbial mimetics, may yield additive or synergistic effects. Importantly, these combinations should be informed by the tumor’s spatial and immunological architecture, guided by single-cell technologies, spatial profiling, and machine learning–based modeling. Such integrative frameworks can enable dynamic mapping of TAN/NETs behavior, uncover therapeutic vulnerabilities, and facilitate patient-specific treatment design. In conclusion, while significant progress has been made in uncovering the roles of TANs and NETs in tumor progression, numerous scientific, clinical, and technological challenges remain. A concerted effort combining basic research, translational innovation, and precision oncology is required to fully realize the potential of neutrophil-directed immunotherapy in solid tumors.

## Methodological advances

As our understanding of TANs and NETs continues to evolve, future progress will rely heavily on the convergence of emerging technologies, integrative multi-omics platforms, and translational research. These elements are critical for unraveling the spatiotemporal heterogeneity of TANs and NETs, uncovering clinically actionable targets, and optimizing neutrophil-directed immunotherapeutic strategies in oncology.

### Technological innovations for spatiotemporal profiling

Recent advances have significantly expanded our capacity to dissect the complexity of TANs in situ. scRNA-seq has become a cornerstone of cellular heterogeneity research, enabling the dissection of neutrophil subtypes and differentiation trajectories across diverse tumor types. For instance, scRNA-seq analysis of 17 cancer types revealed the phenotypic diversity and plasticity of TANs, offering mechanistic insights into their roles in tumor progression and immunity [31]. In pancreatic cancer models, scRNA-seq combined with InfinityFlow identified three TAN subsets (T1–T3), among which T3 exhibited pro-angiogenic features and prolonged survival, suggesting its potential as a therapeutic target [30]. Despite its unparalleled ability to resolve cellular heterogeneity, scRNA-seq inherently lacks spatial context due to the requirement for tissue dissociation into single-cell suspensions, thereby precluding the direct observation of TAN localization and spatiotemporal dynamics within the TME [302]. To overcome this limitation, spatially resolved technologies such as mIHC and imaging mass cytometry have been employed to map TAN infiltration patterns and immune interactions in situ.

By employing fluorescence-labeled antibodies against neutrophil-specific markers, mIHC allows direct visualization of TAN localization and distribution within tissue sections through fluorescence microscopy, thereby revealing distinct infiltration patterns in tumor margins, cores, and stromal regions [31]. mIHC has successfully identified TAN infiltration patterns, such as HLA-DR⁺ neutrophils co-localized with T cells in peritumoral regions, and CD15⁺ neutrophils enriched in hepatocellular carcinoma stroma [5, 31]. When complemented by imaging mass cytometry, which enables simultaneous detection of over 30 protein markers at subcellular resolution, providing powerful platforms for spatial mapping of TAN subsets and their interactions with other immune or stromal components within the TME. In a study involving 204 lung cancer patients, this technique enabled high-resolution profiling of neutrophil distribution and phenotypic context [32]. However, the utility of mIHC is constrained by limited multiplexing capacity and reliance on predefined marker panels, potentially overlooking functionally diverse or spatially rare neutrophil states, limiting its ability to reflect the full spectrum of neutrophil functions and distributions [87]. The use of combinatorial antibody staining, such as CD66b to identify TANs and CD34 to distinguish intravascular from extravascular localization, can improve spatial resolution, but remains insufficient to capture dynamic changes in neutrophil behavior or migration [5, 89, 109].

The rapid evolution of spatial omics technologies has accelerated the demand for integrated spatial multi-omics approaches capable of coupling molecular profiling with preserved tissue architecture to decode complex physiological and pathological processes. Imaging-based spatial omics platforms achieve this by using oligonucleotide probes for in situ hybridization of RNA or antibodies for protein detection, combined with fluorescence or isotopic labeling and high-resolution imaging to visualize the spatial distribution of target molecules [303]. One representative technique is co-detection by indexing (CODEX), which enables high-dimensional spatial profiling through the simultaneous visualization of numerous protein markers [304]. This technology facilitates detailed mapping of tissue organization and provides novel insights into the TME, aiding in deciphering spatial biological complexity. For example, the integration of CODEX with multi-omics analyses has been employed to delineate the spatial architecture of small-cell lung cancer, uncovering distinct cellular and microenvironmental heterogeneity within the TME [305]. Despite its strengths, CODEX relies on multiple iterative cycles of hybridization, imaging, and signal stripping, and currently lacks the capacity for simultaneous co-detection of RNA and protein targets [303], posing a limitation for truly integrative spatial transcriptomic–proteomic analysis.

Spatial transcriptomics offers an orthogonal strategy for characterizing TANs by preserving tissue architecture during transcriptomic profiling, thereby enabling the integration of gene expression data with spatial localization. Despite technical limitations, such as low RNA abundance and short transcript half-lives in neutrophils, recent studies have demonstrated its utility when combined with scRNA-seq. In pancreatic ductal adenocarcinoma, for example, a study integrating scRNA-seq, ATAC-seq, and spatial transcriptomics revealed that both immature and mature neutrophils undergo irreversible epigenetic, transcriptional, and proteomic reprogramming upon tumor infiltration. These neutrophils converge into a distinct, terminally differentiated dcTRAIL-R1⁺ subset localized to hypoxic and glycolysis-active tumor cores. This subpopulation exhibits a pro-angiogenic gene signature that contributes to disease progression [30]. Such spatial-functional stratification underscores the power of spatial transcriptomics to define microanatomical neutrophil niches and uncover context-dependent therapeutic vulnerabilities.

Microdissection-based spatial omics isolates regions of interest through laser capture or manual dissection for downstream multi-omics analyses. While this approach offers high tissue specificity, it is inherently limited by its inability to interrogate the entire tissue landscape and by its relatively low spatial resolution [303, 306]. Representative platforms include Laser Capture Microdissection and GeoMx Digital Spatial Profiling. For instance, integration of gene expression profiling with immune cell spatial resolution using laser capture microdissection in triple-negative breast cancer uncovered key immunological features of the TME, offering insights into therapeutic targeting strategies [307]. Similarly, in metastatic prostate cancer, GeoMx was used to quantify transcript and protein levels across spatially distinct metastatic sites, enabling refined classification of tumor phenotypes and spatially resolved assessment of intratumoral heterogeneity [308]. The 10x Genomics Visium platform represents a major advancement in spatial transcriptomics, enabling transcriptome-wide analysis across entire tissue sections via spatially barcoded oligonucleotides. This approach permits high-resolution mapping of the full transcriptome in a spatially resolved manner [309–311]. A recent study utilizing Visium HD technology profiled five human colorectal cancer specimens and generated single-cell–level whole-transcriptome maps. It identified spatially distinct macrophage subpopulations with opposing tumor-promoting and tumor-suppressive functions, further validated by spatial correlation with in situ gene expression signatures [311].

In summary, current imaging-based spatial omics technologies offer subcellular spatial resolution but are constrained by limited throughput and molecular coverage. In contrast, sequencing-based spatial transcriptomics enables high-throughput transcriptome-wide profiling, albeit with comparatively lower spatial resolution. These complementary strengths and limitations underscore the pressing need for next-generation spatial omics platforms that can simultaneously achieve high spatial resolution and high molecular throughput, thereby enabling comprehensive and precise spatial characterization of complex tissue ecosystems.

### Emerging analytical and imaging paradigms

To fully realize the potential of these spatial technologies, advanced analytical frameworks are essential. Artificial intelligence (AI) and machine learning algorithms are increasingly being applied to high-dimensional spatial datasets, facilitating automated cell-type classification, spatial domain segmentation, and inference of intercellular communication networks. For example, deep learning models trained on multiplexed imaging or transcriptomic data can identify rare neutrophil phenotypes or predict TAN-mediated immunosuppressive niches across tumor regions. These tools enable the construction of predictive models linking spatial neutrophil behavior to therapeutic response, providing a basis for rational patient stratification and precision intervention design.

In addition, intravital imaging techniques, such as multiphoton microscopy and real-time video detection [275, 308, 312], offer a dynamic window into TAN behavior within live tumor models. These technologies allow visualization of neutrophil trafficking, intratumoral migration, and interactions with tumor or stromal cells in response to therapeutic perturbation. By capturing cellular dynamics in vivo, intravital imaging complements static spatial atlases and provides temporal insight into how TAN subsets adapt to changing microenvironmental cues, including hypoxia, chemokine gradients, or immune checkpoint blockade, etc.

Looking ahead, the integration of multi-omics platforms, including transcriptomics, proteomics, metabolomics, and epigenomics, with spatial and temporal profiling technologies promises to redefine our understanding of TAN and NETs biology. Multi-modal datasets can be harmonized using AI-assisted frameworks to uncover master regulatory nodes, cell-state transitions, and tissue-level immune architectures [313–315]. These systems-level approaches will be instrumental in identifying combinatorial vulnerabilities and designing context-specific therapeutic strategies that address the dynamic and multifactorial nature of neutrophil involvement in cancer.

### Translational applications and the path to precision oncology

The clinical translation of neutrophil-targeted therapies is gaining momentum, with several candidate strategies progressing from bench to bedside. Notably, agents targeting the CXCR1/2 axis, such as navarixin and SX-682, are under active investigation, including in combination with ICIs for advanced non-small cell lung cancer (NCT03473925) [262, 263]. Parallel efforts are evaluating inhibitors of NET formation, including DNase I, PAD4 inhibitors such as Cl-amidine and GSK484, as well as metabolic modulators aimed at disrupting the bioenergetic requirements of NETosis [60, 253]. These approaches must be coupled with biomarker-driven trial designs to stratify patient populations, monitor pharmacodynamic responses, and define optimal therapeutic windows. Emerging biomarkers, including neutrophil-derived extracellular vesicles, NETs-associated molecules, and spatially resolved expression signatures, hold promise for guiding treatment selection and evaluating efficacy.

A key translational challenge lies in preserving the physiological functions of neutrophils in host defense while selectively attenuating their tumor-promoting activities. For example, microbial adjuvants or cytokine reprogramming strategies may shift TANs toward an antitumor phenotype but require careful calibration to avoid systemic inflammation or immune-related toxicity [275]. To address these complexities, integrative, cross-disciplinary strategies are essential. The convergence of genomics, epigenomics, proteomics, and metabolomics with spatially resolved technologies and bioinformatic modeling is enabling high-resolution mapping of neutrophil states, plasticity, and functional outputs. In particular, integrating transcriptomic data with spatial proteomics and intracellular signaling networks may reveal critical regulatory nodes amenable to therapeutic intervention.

AI and machine learning are poised to play a transformative role in this landscape. These tools can decipher high-dimensional multi-omic datasets, predict neutrophil phenotypic transitions, and uncover dynamic cell–cell interactions within the TME. Such computational advances will be indispensable for identifying druggable targets, modeling treatment responses, and guiding rational combination strategies. Together, these technological innovations are redefining the future of neutrophil-focused oncology. By bridging cellular heterogeneity, spatial context, and temporal dynamics, they provide unprecedented insight into the roles of TANs and NETs across tumor types. The integration of spatial multi-omics platforms with advanced analytics is expected to accelerate the translation of neutrophil-targeted strategies, from preclinical discovery to precision immunotherapy.

As the field advances toward a framework of spatiotemporally resolved precision oncology, TANs and NETs are poised to emerge as both predictive biomarkers and therapeutic targets across diverse malignancies. Realizing this potential will require a sustained commitment to interdisciplinary collaboration, iterative validation in clinically relevant models, and the development of patient-specific therapeutic algorithms that reflect the dynamic complexity of neutrophil biology.

## Conclusion

The spatiotemporal heterogeneity of TANs and NETs represents a fundamental axis in the regulatory architecture of the TME, exerting profound effects on tumor progression, immune modulation, and therapeutic responsiveness. In this review, we have systematically examined the dualistic roles, spatial compartmentalization, and dynamic transitions of TANs and NETs, with particular emphasis on their functional evolution across tumor stages and microanatomical niches. We further dissect the key molecular and microenvironmental determinants shaping TAN behavior and outline rational strategies for leveraging this knowledge in next-generation immunotherapeutic design.

TAN heterogeneity manifests not only through functional plasticity, exemplified by the interconversion between anti-tumorigenic and pro-tumorigenic phenotypes, but also through complex spatial and temporal distribution patterns. Notably, peritumoral TANs frequently display heightened tumor-promoting activity, contributing to local invasion and distant metastasis, whereas intratumoral TANs may adopt more immune-modulatory roles [88, 312]. Simultaneously, NETs actively remodel the TME through the release of proteolytic enzymes (e.g., MMPs), ROS, and DNA scaffolds, reshaping both its structural integrity and immune landscape [111, 239]. These spatiotemporally constrained functions underscore the necessity for context-dependent targeting of TAN and NET activity throughout cancer progression.

The distribution of TANs is tightly regulated by a diverse array of chemokines, cytokines, and metabolic cues within the TME. We provide an integrated synthesis of TAN distribution patterns across various tumor entities, elucidating conserved and tumor-specific regulatory pathways that drive TAN recruitment and polarization. These insights not only enhance mechanistic understanding but also inform the rational identification of therapeutic entry points. In this context, we review emerging interventions, including chemokine receptor blockade, cytokine modulation, and metabolic reprogramming, that hold promise for reshaping TAN behavior and augmenting the efficacy of established immunotherapies. A comprehensive understanding of TAN dynamics will be indispensable for designing precision-guided combination regimens tailored to individual TME architectures.

Despite significant progress, many aspects of TAN and NETs biology remain incompletely understood, particularly in relation to their spatiotemporal heterogeneity. This unresolved complexity may underlie several key challenges in current immunotherapy paradigms, including treatment resistance and heterogeneous response profiles [21, 57, 86]. To address these limitations, we highlight a suite of cutting-edge technologies, ranging from scRNA-seq and spatial transcriptomics to mIHC and high-resolution imaging, that enable granular characterization of neutrophil states in situ. Integrative multi-omics approaches, coupled with computational modeling and AI, are poised to generate comprehensive atlases of TAN/NETs dynamics that capture their behavior in both space and time.

Looking ahead, neutrophil-targeted therapies guided by spatiotemporal profiling have the potential to overcome current limitations in cancer immunotherapy. By elucidating the context-specific roles of TANs and NETs, and by aligning therapeutic strategies with the spatial and temporal logic of the TME, these advances may pave the way toward truly personalized immuno-oncology. Ultimately, translating neutrophil-directed interventions into clinical reality will require sustained interdisciplinary collaboration, biomarker-informed patient selection, and the integration of technological innovation with therapeutic development.

## Data Availability

No datasets were generated or analysed during the current study.

## Abbreviations

TME: Tumor microenvironment
TAN: Tumor-associated neutrophil
ROS: Reactive oxygen species
VEGF: Vascular endothelial growth factor
ECM: Extracellular matrix
NET: Neutrophil extracellular trap
EMT: Epithelial–mesenchymal transition
TLS: Tertiary lymphoid structures
scRNA-seq: Single-cell RNA sequencing
CAF: Cancer associated fibroblast
MPO: Myeloperoxidase
MMPs: Matrix Metalloproteinases
SCF: Stem cell factor
PAD4: Peptidyl arginine deiminase 4
G-CSF: Granulocyte colony-stimulating factor
Tregs: Regulatory T-cells
ICIs: Immune checkpoint inhibitors
H3Cit: Citrullinated histone H3
HIF: Hypoxia-inducible factor
TAMs: Tumor-associated macrophages
DCs: Dendritic cells
NK cells: Natural killer cells
AGR2: Anterior gradient protein 2
TIMP-1: Tissue inhibitor of metalloproteinase-1
mIHC: Multiplex immunohistochemistry
sRAGE: Soluble receptor for advanced glycation end-products
CODEX: Co-detection by indexing
AI: Artificial intelligence
